# Physical Modification of Starch Digestibility: A Comprehensive Review and AI-Assisted Qualitative Knowledge Extraction

**DOI:** 10.3390/foods15173137

**Published:** 2026-09-03

**Authors:** Moshit Yaskin Harush, Carmit Shani Levi, Uri Lesmes

**Affiliations:** Laboratory of Chemistry of Foods and Bioactives, Department of Biotechnology and Food Engineering, Technion–Israel Institute of Technology, Haifa 3200003, Israel; moshityaskin@campus.technion.ac.il (M.Y.H.); shanilc@bfe.technion.ac.il (C.S.L.)

**Keywords:** process parameterization, AI, annealing, heat moisture treatment, autoclaving, readily digestible starch, resistant starch

## Abstract

Starch modification through physical processing represents a promising “green” strategy to enhance food functionality and nutritional quality while meeting clean-label demands. This review offers a critical overview of the structural and nutritional impacts of physical modifications coupled with an exploratory use of artificial intelligence (AI)-assisted literature for screening and mining. Focused on literature published between 2001 and 2026, a traditional human-led systematic search identifies eligible studies that examined the effects of three major commercially relevant modification techniques—annealing (ANN), heat–moisture treatment (HMT), and autoclaving—on rapidly digestible starch (RDS), slowly digestible starch (SDS), and resistant starch (RS), with emphasis on resistant starch type III (RS3). Among the evaluated techniques, autoclaving, particularly when followed by retrogradation, appears to offer the greatest potential for increasing RS relative to the original native starch. However, its effectiveness is strongly dependent on the raw material, with substantial gains arising from its amylose content, while waxy starches may show little improvement or even a reduction in RS. ANN tends to produce milder and more variable effects, often shifting starch from RDS toward SDS. HMT generally reduces RDS and increases SDS, making it a promising approach for attenuating glycemic responses. However, the extent of these changes can vary substantially depending on the starch source, amylose content, moisture level, and processing temperature. Overall, the work revisits the potential of physical processing as an avenue for starch engineering while underlining a pressing need for standardized workflows, harmonized methodologies, and unified protocols for quantification of starch digestibility, namely, of RS levels. Lastly, the review exemplifies AI can accelerate preliminary literature identification and synthesis yet highlights gaps in AI aptitudes for independent quantitative integration that still maintains a need for thorough human-driven validation.

## 1. Introduction

Nowadays, the food industry seeks to deepen its abilities to harness processing to enhance and extend food safety, shelf life and wholesomeness. From the nutritional perspective, processing has been explored as a tool to influence nutritional profile of foods as well as direct their digestive fate. To this end, starch, a natural abundant and affordable macronutrient, is widely used across various industries [1] and highly affects bodily responses, like glycemic responses, satiety and satiation [2,3]. Strach is also a highly versatile food component and energy source whose versatility arises from its unique structure, composed primarily of a-glucans intertwined in complex architectures: mixtures of amylose and amylopectin [4].

Native starches often have techno-functional limitations, such as poor solubility, viscosity loss, syneresis, and a high tendency for retrogradation. As a result, the global market for starch modification reached up to USD 17.15 billion in 2026 and is expected to expand at an average annual growth rate of 2.67% over the 2026–2031 period [5]. A range of methods are employed to obtain starches with the desired properties for various industrial applications, including chemical, enzymatic, and physical approaches [6,7,8]. Such modifications are frequently used to extend food shelf life and safe use but not devoid of disadvantages such as changes in food matrix properties, palatability and compromised nutritional properties. To this end, starches are categorized into three types according to their digestibility: rapidly digestible starch (RDS, measured after 20 min of digestion), slowly digestible starch (SDS, measured after 120 min of digestion) and resistant starch (RS, defined as the undigested fraction). The latter persists into the colon where it undergoes fermentation by the microbiota [9,10]. Thus, many studies seek to increase RS content of starchy foods as well as change the ratio between RDS/SDS/RS.

Chemical modification techniques focus on starch derivatization and are widely used due to their cost-effectiveness and simplicity of application [11]. These include chemical substitution, degradation, cross-linking, oxidation, esterification, and acetylation [12,13,14]. Such techniques yield significant effluents that require recycling and can pose environmental risks as well as dampen the food sectors’ demand for “clean labels”. Consequently, physical modifications are increasingly preferred as “cleaner” or “greener” methodologies for starch modification [15,16,17]. These involve heat treatment (dry and moisturized), high pressure processing, ultrasonic, pulse electric field, annealing, and extrusion to change starch structure or physical state without altering its chemical composition [18]. These processes can alter starch’s physical properties such as granule size, crystallinity, gelatinization temperature, water absorption capacity, and the RDS/SDS/RS ratio. In fact, RS is an advocated prebiotic with associated health benefits, including improved insulin sensitivity, lowered risk of colon cancer and cardiovascular disease, and elevated feeling of satiety [19,20,21], thus stimulating efforts to boost RS levels in foods. However, most commercial food contains low levels of RS which are well below levels needed for physiological effects or the current recommendations for dietary fiber intake [22,23]. Thus, it is essential to systematically develop methods to increase its content in food products as well as in the starch itself, as a staple ingredient [22].

To date, numerous studies have examined how physical modifications affect starch properties and digestibility. However, the large volume of data generated over the years, together with limited methodological harmonization, has constrained the ability to translate vast volumes of data into a “cook and look” approach towards rational design of processing and formulation strategies [24]. This review therefore revisits studies on starch physical modifications to identify processing and formulation determinants that drive starch digestibility. Extending the past focus on starch techno-functionality, emphasis is drawn to commercially relevant scenarios aimed at enriching foods with RS while considering the balances among RDS, SDS, and RS.

Given the soaring availability of artificial intelligence (AI) tools, there is a growing concern that AI tools could compromise the skills of professionals rather than advance knowledge and professional capacities [25]. Thus, this review is further supported by an exploratory evaluation of their simple and practical use in literature mining and qualitative knowledge extraction. Specifically, AI tools were deployed to identify, categorize and organize the diverse food science literature to recognize recurring research themes, metrics and key starch determinants. These outputs were qualitatively assessed against a parallel human-led process. Accordingly, this work offers two complementary contributions: first, a critical synthesis of scientific literature from the past 25 years to help underpin effective techniques and process metrics for starch physical modification; and second, an exploratory assessment of AI-assisted qualitative knowledge extraction, including its utility and limitations. Overall, the review aims to support food engineering strategies for enhancing SDS and RS levels in starchy foods, with potential implications for improved health outcomes.

## 2. Mining for Studies on Physical Starch Modification—Literature Survey Strategy

### 2.1. Materials and Methods to Identify Relevant Studies

The literature review and data analysis carried out herein followed several steps, that is, article identification and screening, data extraction, and integration. To this end, the survey conducted targeted research articles on physical modification of starch via thermal processes, specifically annealing (ANN), heat-moisture treatment (HMT), and autoclaving, published during 2001–2026 (autoclave 2001–2025, HMT 2002–2025, ANN 2005–2026) and listed in Google Scholar and/or ISI Web of Science.

### 2.2. Human-Driven Versus AI-Assisted Article Search

**Figure 1:**
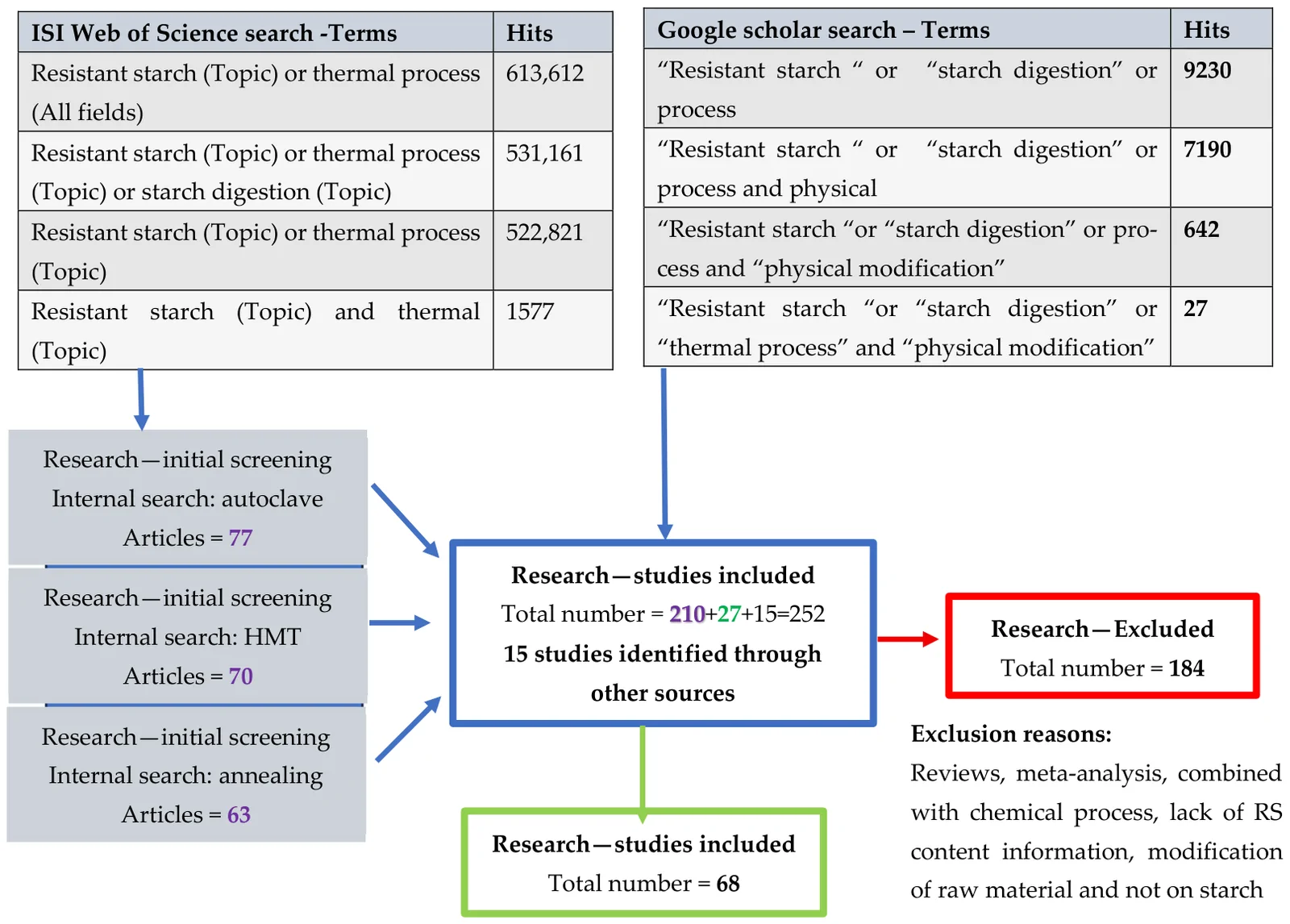
**Flowchart of the systematic literature search strategy and selection process.** The diagram illustrates the initial search questions and total hits retrieved from the ISI Web of Science and Google Scholar databases, followed by internal screening (autoclaving, HMT, and ANN). A total of 252 studies were initially identified (including 210 from database screening, 27 from specific refined criteria, and 15 from alternative sources). After applying exclusion criteria (*n* = 184) due to reasons such as review papers, lack of RS content data, etc., 68 final studies were included in the analysis.

A thorough data analysis was conducted to examine how different starch modification techniques influenced levels of RDS, SDS and RS and the determinants thereof. The materials needed for the data analyses and literature review were research articles targeting starch modification by thermal physical techniques containing qualitative and/or quantitative data of the values of RDS, SDS, and RS prior and post modification. In fact, the AI component in this study was used for mining and qualitative knowledge extraction rather than for autonomous quantitative synthesis. This qualitative approach was adapted, assuming expected simplistic use of AI tools by food professionals inexperienced in AI and professional use of LLMs as well as some identified limitations of the AI and the data presented in the scientific literature. To ensure an accurate and systematic screening process, inclusion and exclusion criteria for identified studies were established, as detailed in Figure 1. This work is presented as a critical narrative review of the literature on physical starch modification rather than as a meta-analysis review. Therefore, a formal PRISMA checklist was not applied. Nevertheless, the search strategy, search strings, database results, and inclusion/exclusion criteria are transparently described in Figure 1.

The inclusion criteria included articles published in English between 2001 and 2026 that used native starch as the initial substrate and applied thermal–physical modification techniques under varying experimental parameters. The review was limited to three modification methods: ANN, HMT, and autoclaving. Data extraction focused on both starch characteristics and processing conditions. Starch-related information included the botanical source and amylose content. Process-related variables included the water-to-starch ratio, equilibration conditions following starch hydration, number of processing cycles, heating temperature, heating duration, retrogradation conditions, and the number of repetitions.

Based on these data, the change in RS content was calculated by comparing RS values prior and post modification treatment. In addition, information regarding RS quantification methods and drying procedures was collected, as these parameters varied considerably among studies and may have influenced the magnitude of the reported RS alterations. It should be noted that not all extracted parameters were applicable to each modification technique. For example, multiple processing cycles are characteristic of autoclaving treatments but are uncommon in ANN and HMT processes. Similarly, retrogradation is frequently incorporated as part of autoclaving protocols but is generally not applied in ANN or HMT treatments. Further, the study exclusion criteria dropped studies that used pre-treated starch, dual or combined processes (e.g., enzymatic and thermal processing), had experimental flaws (e.g., lack of proper experimental controls such as untreated sample), non-experimental literature reviews, and articles with missing or incomplete data on resistant starch content. The absence of RDS and SDS data did not constitute an exclusion criterion; however, when such data were available, they were also examined and discussed in this study.

This initial ISI web of science search yielded 1577 studies, but further refinement based on the production method (autoclave, HMT or annealing) trimmed the database to 210 articles. These were augmented with 27 papers identified via Google Scholar and 15 articles identified through other sources. This generated a literature base of 252 full-text articles that were subsequently refined to a total of 68 studies following removal of duplicates and exclusion of studies (according to the criteria detailed in Figure 1).

Concomitantly, ChatGPT (free version, accessed on 11 January 2026) and Perplexity (Pro version, accessed 11 January 2026) Large Language Models (LLMs) were prompted in January 2026 with the following single prompt, focusing on autoclaving: “Act as a scientific literature review assistant specialized in food chemistry and starch science. Systematically identify, analyze, and synthesize peer-reviewed research articles on the modification of starch using **physical and thermal methods** to increase **resistant starch (RS) content**. Focus on methods such as heat–moisture treatment, annealing, autoclaving–cooling cycles”. This prompt yielded a rapid response with two literature datasets: one with a list of 16 research papers (from ChatGPT) and another with a list of 15 articles with direct links or Digital Object Identifiers (DOIs) identified by Perplexity. Screening the references revealed two internal-list duplicities and two inter-list duplicities, therefore yielding a limited aggregate result of 28 articles compared to the human-driven list generated via ISI Web of Science and Goggle Scholar that yielded 210 articles.

The sharp contrast between the 210 scientific articles identified by manual search and the list of 28 articles generated by AI emphasizes the possible gaps and limitations between the tedious, time-consuming human search with a simple, straightforward, one-time conversational AI query. On one hand, laborious manual search retrieved a higher number of publications through access to free and subscribed resources compared to conversational AI interfaces that operate primarily within open-access networks and lack built-in programming integration into subscription-based, indexed academic databases. Moreover, LLMs like ChatGPT and Perplexity are non-deterministic which means their outputs can vary even with identical prompts presented at different times. Therefore, this study highlights the gaps between human and AI-driven literature mining, illustrating the constraints of AI-based literature mining. Rather than a reproducible systematic review framework, the AI component here is used as an exemplary case for a simplistic use of AI by food professionals that are not AI tech-savvy. This emphasizes the critical need for human-expert inspection and increasing AI savviness of food professionals; all automated findings in this work were thoroughly cross-referenced and manually verified to guarantee scientific merit. Furthermore, the present evaluation was based on a single, standardized prompt for each platform, reflecting how a non-AI savvy food professional might use an LLM for an initial literature search, rather than the more iterative approach typically used by an expert. This approach does not capture the full potential of LLM-assisted literature searching, which can benefit from an iterative process in which prompts are refined, additional or missed references are requested, and the search is progressively improved through multiple rounds of interaction. Therefore, the AI-assisted search results presented here should be viewed as simple exemplars of how LLMs perform when used with a single prompt, rather than as a measure of their full potential. Future studies and applications of AI should compare this simple approach with more interactive and refined search strategies, including multiple rounds of prompting and, where possible, LLM tools connected to scientific databases. Such comparisons would provide a clearer understanding of how effectively AI can support systematic literature searching in food science.

### 2.3. AI-Assisted Harvesting of Articles

The overall goal of this review was to revisit starch modifications and integrate the available evidence from the literature to generate an overarching trajectory and/or identify optimal conditions to maximize RS content. The use of AI in academic literature searches and data integration offers clear advantages that are particularly appreciable during the initial stages of a literature review. In these stages, AI-based tools facilitate rapid identification of relevant concepts, processing methods, and process parameters that are often studied in food research. This may help unveil research trends and predominant experimental approaches, such as the frequent use of autoclaving–cooling cycles to increase RS content, thereby helping researchers navigate the literature more efficiently and focus on individual studies. When used with verified open-access sources, AI can summarize complex experiments and results more efficiently, reducing search time, screening, and focusing discussions according to relevant subjects that emerge from an AI review. On the contrary, AI-based search lacks the ability to critically distinguish between study differences, delve into nuances of experimental design and analytical approaches. For example, AI commonly compares reported RS values without adequately accounting for methodological differences (such as method for RS determination or process parameters like temperature profile, number of heating–cooling cycles, drying method, and storage conditions) or alterations from baseline measurements, potentially leading to misleading interpretations regarding process efficiency or superiority. Thus, conclusions based on AI-assisted comparisons may oversimplify outcomes or include invalid comparison of experimental evidence. This is augmented by the limited ability of AI searches to access paywalled or less-cited scientific journals, compromising the comprehensiveness of literature reviews or identifying underlying mechanistic insights. Importantly, AI has limited ability to critically evaluate experimental design and analytical validity. Thus, variations in analytical methods to determine RS (e.g., Englyst method versus AOAC protocols), sample identity (e.g., botanical origin of the starch), preparation procedures, or the absence of native starch baselines can substantially dampen reported RS values and the ability to synthesize studies into a unifying conclusion or trend. This is particularly concerning as a recent review clearly voiced a need to calibrate and standardize a unified method for assessing RS values in a comprehensive update on the underlying modulation mechanisms of starch digestion [24].

Overall, this exploratory, straightforward single-prompt evaluation suggests that AI-based searching can provide rapid access to relevant information from openly available sources, but with more limited coverage than a human-led search. The human-led approach enabled broader searching, access to specific resources, and a more comprehensive assessment of the relevance, methodological details, and intricate significance of the identified studies. Ultimately, AI appears to be most effective as a complementary tool to extend and intensify critical human reading. It is also important to note that AI-based search did not lead to dead ends or broken links; neither did the human-driven search.

**Figure 2:**
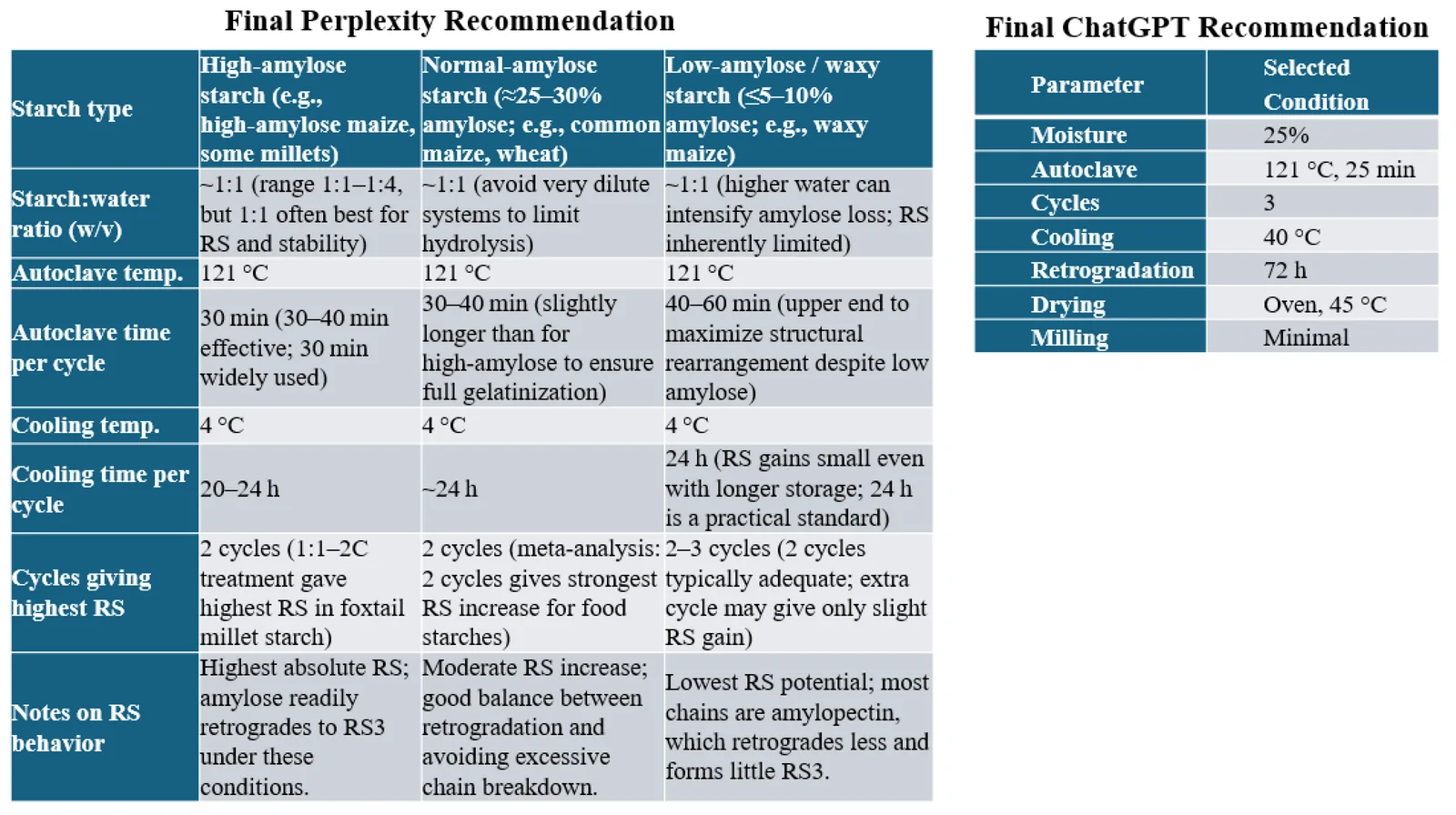
Summary of raw, unverified AI-generated processing recommendations for increase in RS content prior to human-expert validation. Formatting of outputs in table format was generated independently by both the freely accessible Standard version of Perplexity and ChatGPT 5.3.

Next, both AI platforms were prompted to “*summarize the parameters reported across different studies and highlight the values recommended to maximize RS content based on this comparative analysis*”. This sought to explore the potential of AI to offer an overview and extract valuable determinants applicable to food engineering with RS as the key outcome. Figure 2 shows the raw, unedited recommendations generated by the AI tools. It is important to note that these outputs are not presented as established evidence in food science but rather as a baseline for further evaluation. To reframe and validate these recommendations, each parameter generated by the AI tools was systematically cross-referenced against the human-reviewed literature. This human validation process revealed significant variances and deviations from the initial suggestions provided by the AI tools.

For example, the literature suggested that using an autoclave at a **temperature** of 145 °C [26] resulted in a higher RS yield compared to 121 °C, the key temperature that was suggested by both ChatGPT and Perplexity. Regarding process **duration**, the AI-generated printout did not align with the findings obtained through human-led research and analysis. For example, AI platforms suggested a duration of 30–40 min while studies like Onyango et al. [27] yielded high RS within a relatively short process window. Their study indicates that exceeding optimal points (e.g., beyond 45 min) decrease RS yield, thus challenging the pragmatics of setting the autoclaving duration to 40 min, as suggested by AI. Similarly, the non-deterministic nature of AI yielded inconsistent parameters of **autoclave–cooling cycles** and **moisture content**. Moreover, both AI tools disregarded starch pretreatment, which may affect RS formation, as previously shown [28]. Thus, AI was found to be a robust and simple tool to expedite search and offer opportunities for supervised integration of studies that should be handled with caution. This could facilitate qualitative identification of determinants (such as retrogradation time, retrogradation temperature, moisture content) or overall trends but not to be used as an unrestrained or unsupervised replacement of professional researchers.

## 3. Integrative Mining of Starch Parameters and Processing Determinants

Native starches often exhibit health-related and technological-related benefits stimulating the use of physical modification techniques in commercial processing in a quest to tailor their functionalities without chemical derivatization. This review focused on adapting the current state of the art for evidence-based physical modifications of starch while attempting to harness an AI-based approach. The studies identified herein highlight that the content of RS, specifically retrograded type III starch (RS3), is significantly influenced by the interplay of multiple processing and formulation variables. To this end, the botanical origin of the starch and the size of its granules have been found to be the most critical factors in determining susceptibility to enzymatic attack and the breakdown of starch molecules [24,29]. For instance, cereal starches like rice and corn often possess granules with surface pores and channels that facilitate enzyme infiltration while tubers like potato lack these fissures, thereby proving to be more resistant to digestion [29]. Relevant studies further establish that amylose content of the raw material is possibly the strongest determinant of RS3 formation believed to arise from intricate amylose–amylose and amylose–amylopectin interactions which lead to more compact starch structures with reduced enzyme accessibility. Accordingly, a strong positive correlation has been reported between high amylose levels in starch and process-induced RS formation [29,30]. HACS typically yields significantly higher RS content than normal or waxy corn starch attributable to its tendency to easily form stable, double-helical structures made from closely aligned linear biopolymer chains through hydrogen bonding [31]. In contrast, waxy starches, poor in amylose, tend to produce very low amounts of RS3 upon processing [26,32,33]. Altogether, amylose content, determined mostly by the botanical origin of the starch, is inversely related to its digestibility associated with increased SDS and RS, and decreased RDS [34]. However, it is important to note that high-amylose starches tend to exhibit higher absolute RS contents, whereas lower-amylose starches show greater relative process-induced increases in RS compared to their native counterparts [35].

### 3.1. Process Parameters Influencing RS Content

While intrinsic starch factors are detrimental to starch functionality, they pose inherent challenges that might be tackled through processing techniques and conditions that facilitate diverting the interplay between RDS, SDS and RS. In the face of the reviewed body of scientific literature, this study focused on three prominent commercially viable techniques: ANN, HMT, and autoclaving. Due to pragmatic considerations, inter-comparison of processes while operating in the same manufacturing conditions could not be attained.

This is because each modification technique is defined by a distinct combination of temperature, moisture content, pressure, and treatment duration that collectively determines its mode of action.

First, unlike other hydrothermal methods, ANN is a hydrothermal method that uses higher moisture content, either excess water (>65% *w*/*w*) or intermediate water content (40–50% *w*/*w*) and temperatures exceeding the glass transition temperature (*Tg*) while below the starch onset temperature of gelatinization (*To*), and its duration can vary from hours to days. This process alters the physicochemical properties of starch without destroying the granule by improving its crystalline structure and promoting interactions between starch chains (such as amylose–amylose and amylose–amylopectin interactions) under mild conditions and prolonged durations. During annealing, starch undergoes reorganization, resulting in a more arranged configuration and generation of RS [15,17,36,37,38,39,40]. In search of evidence that could facilitate mild starch processing, this work offers a summary of studies that report the impact of annealing on digestible and indigestible fractions of various starches (Table 1 and Supplementary Table S1).

These studies indicate treatment temperature is critical and set not to exceed the onset temperature (*To*) of gelatinization (typically 50–55 °C or 10–15 °C below the *To* of the specific starch). However, if the ANN temperature is too high, it may disrupt starch crystallites or increase porosity, leading to higher fractions of RDS at the expense of lower RS content.

For example, Chung et al. [41] observed that starch annealed at 10 °C below the onset gelatinization temperature (*To*-10 °C) compared to annealing at 15 °C below the onset temperature (*To*-15 °C) improved RS content of corn, pea, and lentil starches and attributed it to the improved crystalline structure rendered more resistant to enzymatic hydrolysis. On the other hand, application of annealing to two different Grass pea starch, at *To*-10, significantly decreased the RS content, while increasing SDS levels [42]. Similarly, ANN duration (typically 24–72 h) has been found to have distinct but insignificant effects on RS content [43]. Notably, ANN seems to be implicated in a significant increase in SDS and RS at the expense of reducing RDS levels. However, Table 1 highlights that mixed trends were observed in the relevant studies, and some also emphasized selective effects of heating/cooling cycles [43] and/or moisture content. For example, excess moisture attenuated RS content for four different starches after specific ANN conditions (72 h, 55 °C, 90% moisture) [39].

On the contrary, HMT is considered a more intensive unit operation that involves treatments at low moisture content (less than 35%) but a higher temperature range (80–140 °C), exceeding the glass transition temperature. It is conducted for several hours while still retaining the granular structure without inducing gelatinization [11,38,44,45]. In line with the review’s objective, Table 2 and Supplementary Table S2 summarize relevant studies. Such combined heat and moisture treatments can enhance the flexibility and mobility of the starch chains, leading to disruption and in some cases even breaking of the intermolecular and intramolecular hydrogen bonds within the starch granules [46,47]. Overall, higher moisture levels (within the HMT range) typically correlate with higher RS formation. Again, in the case of HMT processing, the effect of moisture and other process parameters on RS content is not straightforward.

Lastly, autoclaving is considered highly energy intensive and involves treatment of starch slurries (typically at 70–90% moisture), at elevated temperatures (120–150 °C) and high-pressure steam of 1.5–3.0 atm, for controlled durations of 30–120 min. This process has been extensively studied (as detailed in Table 3, and Supplementary Table S3) and found to involve hydration and swelling of starch amorphous regions while the crystalline regions melt and are disrupted (gelatinize); this destruction leads to the leaching of amylose and rearrangement of amylopectin, which leads to the generation of RS during the retrogradation phase [48,49,50,51].

Similar to the previous processes, the identified studies comparing autoclaving at different temperatures indicated processing temperature increased RS content [52]. Again, this trend can be counteracted by other studies, like the study by Soler et al. that showed that this trend was delineated by amylose content. For example, when very high amylose content (around 70%) in starch was examined, RS levels increased as the autoclaving temperature rose from 105 °C to 120 °C, but a further increase in temperature to 135 °C resulted in a reduction in RS content [53]. Additionally, repetitive thermal treatments (heating and cooling cycles) were found to be commonly applied to increase the proportion of RS via autoclaving, typically 3–5 cycles [33,54]. Reviewing the evidence, this trend also differed between waxy and non-waxy starches with waxy starch decreasing RS content as the number of cycles increased [32]. Moreover, a significant proportion of studies dwelled on the importance and impact of starch retrogradation to the buildup of RS.

It is important to note that a small number of individual studies, applying a single internally consistent protocol across several conditions, offer illustrative examples of potential parameter interactions that merit dedicated future investigation. In the study by Rahayu, increasing the autoclaving temperature resulted in higher RS content. At the same autoclaving temperature, extending the retrogradation period from 24 to 48 h further increased the RS content. The combination of these two conditions, higher autoclaving temperature and longer retrogradation, resulted in the greatest RS increase compared with the other temperature and retrogradation-time combinations evaluated [52]. When comparing retrogradation after applying an autoclave process, at a fixed temperature but for a longer duration, RS content generally increased across most of the temperatures examined (−20, 4, 30, 60, and 100 °C), with the exception of 30 °C. In contrast, when the retrogradation time was kept constant while the temperature was varied, an optimal temperature of 4 °C was observed for a 6 h retrogradation period, with RS enhancement decreasing as the temperature increased above or below that value. However, for longer retrogradation periods of 24 and 48 h, the greatest RS increase was observed at 100 °C [27]. When examining the effect of moisture content (80% vs. 90%) on RS formation after autoclaving, higher RS levels were generally obtained at the lower moisture content of 80%. Increasing the number of processing cycles from one to two and three resulted in an overall reduction in RS content, although RS increased progressively with each additional cycle; however, the values remained below those of the native starch. A positive RS response was observed only when the maximum number of cycles evaluated was combined with 80% moisture [33]. These findings are presented as illustrative observations from individual studies rather than as a cross-study synthesis, given the substantial methodological heterogeneity among the protocols, which limits the validity of direct comparisons across the broader body of literature summarized in Table 1, Table 2 and Table 3.

**Figure 3:**
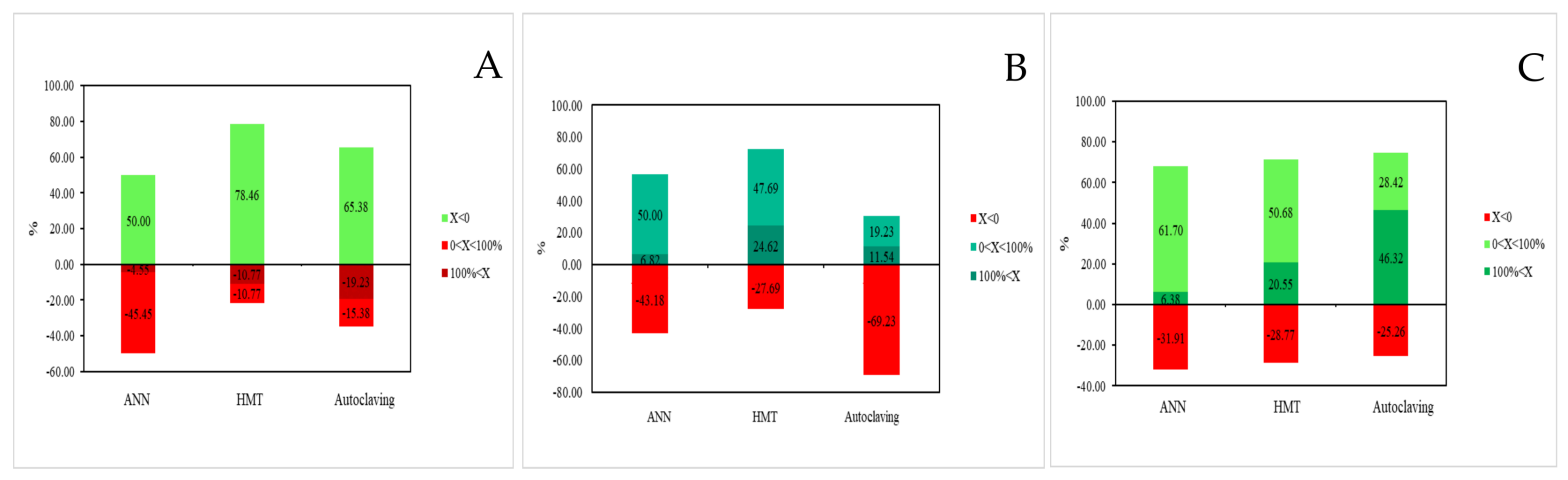
Percentage changes in RDS (noted (**A**)), SDS (noted (**B**)), and RS (noted (**C**)) following different processing treatments. Percentage changes are calculated relative to each study’s native-starch control and should not be interpreted as direct rankings of process efficiency, due to differences in RS assays, drying procedures, moisture basis, and starch sources across studies (see Section 3.2).

In summary, a considerable body of evidence gives note to a mixed trend where processing can boost or compromise the digestibility of starch and its content of RS. Therefore, an additional analysis of the gathered studies was performed to qualitatively assess the magnitude of the processing effects on starch qualities. For this, the processing impact on each starch fraction (RDS:SDS:RS) was color-coded with shades of green indicating increasing levels of the corresponding fraction and red indicating attenuation in their content, based on the percentage of change compared to baseline level of the same starch before processing (as detailed in Table 1, Table 2 and Table 3). Plotting alterations in starch fractions of RDS, SDS and RS (Figure 3) enables the visualization of the trends observed throughout the identified studies. First, this analysis enables a qualitative differentiation between the magnitudes of studies indicating an increase in starch fraction versus those who demonstrated an attenuation, i.e., balancing between the magnitude of green and red bars. Moreover, Figure 3 facilitates a comparison between the various processes of ANN, HMT, and autoclaving. Altogether, Figure 3A,B stress that HMT has an overall positive trend on digestible starch fractions, expressed as a lowering of RDS and increasing SDS levels of starch. Moreover, autoclaving is found to have detrimental effects on the SDS content of starch. Having starch digestibility in mind, one could suggest HMT as a favorable processing pathway towards physical modulation of starch. In contrast, Figure 3C highlights a clear benefit for autoclaving as a pathway to boost RS content of starch. This figure also demonstrates that the most significant effect of autoclaving is a dramatic increase in RS levels (marked in dark green) exceeding 100%, compared to the corresponding unprocessed starch fraction.

It is important to note that the variation between the green and red bars in Figure 3 reflects the strong influence of raw material and processing conditions. None of the processes showed consistently positive or negative effect across all studies. The magnitude, and in some cases even the direction, of the response varied with starch source, amylose content, moisture level, and temperature. Thus, Figure 3 should therefore be viewed as illustrating general trends rather than predicting a specific response for every raw material. To facilitate comparison among the three modification techniques, Table 4 summarizes the recommended processing conditions, expected changes in RDS, SDS, and RS, and the underlying mechanisms of ANN, HMT, and autoclaving. By bringing together the trends presented in Table 1, Table 2 and Table 3 and Figure 3, this synthesis provides a practical reference for selecting and optimizing modification process according to the amylose content of the target starch.

The three processes provide distinct advantages rather than a single “best” approach. ANN is the mildest option, offering a modest shift from RDS to SDS while minimizing the risk of over-processing or undesirable structural changes. It is therefore particularly suitable when gentle, granule-preserving modification is preferred over maximizing RS yield. HMT provides a more consistent single-step approach for achieving a substantial RDS-to-SDS shift and can produce considerable RS increases without the need for multiple processing cycles. This makes it attractive when processing time and energy efficiency are important. Autoclaving can achieve the greatest overall increases in RS. Its benefits increase markedly with the number of cycles. Although autoclaving offers the greatest potential for RS enhancement, it also carries the highest risk of decreasing SDS content and requires careful tailoring of process parameters in order to minimize unwanted SDS content decreases.

### 3.2. Limitations and Challenges When Comparing Studies

A key limitation of the dataset is the methodological heterogeneity across studies, including differences in RS assay methods, drying procedures, moisture basis, and starch sources. Therefore, the percentage changes reported in Table 1, Table 2, Table 3 and Table 4 and Figure 3 should be viewed primarily as within-study indicators of direction and magnitude, rather than as directly comparable rankings across studies.

**Table 1:** Alteration percentage after applying the annealing process.

Comparison Parameters	Starch	Native Starch			Modified Starch			% Alteration (vs. Native Baseline)			Ref
		RDS ± SD	SDS ± SD	RS ± SD	RDS ± SD	SDS ± SD	RS ± SD	RDS	SDS	RS	
**Starch source**	Black 8025 bean starch	22.8 c	34.98 a	36.15 f	24.16 b	24.48 d	43.26 cd	5.96	−30.02	19.67	Simsek et al. 2012 [55]
	Pinto Durango bean starches	25.42 a	27.16 b	41.61 e	13.98 d	22.27 e	58.13 a	−45.00	−18.00	39.70	
**Temp. & Duration**	Castanopsis sclerophylla starch	75.2 ± 1.7 a	18.3 ± 2.7 c	6.5 ± 1.7 a	71.3 ± 0.3 b	23 ± 0.9 a	5.7 ± 0.9 a	−5.19	25.68	−12.31	Shi et al. 2021 [43]
		75.2 ± 1.7 a	18.3 ± 2.7 c	6.5 ± 1.7 a	71 ± 2.6 b	20.7 ± 0.9 ab	8.3 ± 1.9 a	−5.59	13.11	27.69	
		75.2 ± 1.7 a	18.3 ± 2.7 c	6.5 ± 1.7 a	72.5 ± 0.7 b	19.8 ± 1.2 bc	8.8 ± 1.4 a	−3.59	8.20	35.38	
**Starch source**	Sweet potato starch Yulmi -YM	20 ± 2.2	34.2 ± 2.2	45.8 ± 0.0	28.3 ± 0.5	44.8 ± 0.6	26.9 ± 0.1	41.50	30.99	−41.27	Song et al. 2014 [39]
	Sweet potato starch Yeonwhangmi—YHM	22 ± 0.6	35.4 ± 0.4	42.6 ± 0.1	29.3 ± 0.3	45.3 ± 0.8	25.4 ± 0.6	33.18	27.97	−40.38	
	Sweet potato starch from Samyang Genex—SSPS	14.2 ± 0.7	38.5 ± 0.2	47.4 ± 0.9	20.6 ± 0.2	46.5 ± 0.6	32.9 ± 0.6	45.07	20.78	−30.59	
	Commercial sweet potato starch—CSPS	5 ± 0.1	30.9 ± 1.0	64.1 ± 1.0	5.6 ± 0.2	32.2 ± 1.4	62.2 ± 1.2	12.00	4.21	−2.96	
**Starch source**	Tartary buckwheat starch (TBS)	37.6 ± 0.12 a	42.5 ± 0.23 ab	3.27 ± 0.33 b	34.9 ± 0.07 b	44.6 ± 0.05 a	4.75 ± 0.21 a	−7.18	4.94	45.26	Liu et al. 2016 [34]
	Sorghum starch (SS)	36.8 ± 0.23 a	41.9 ± 0.12 b	3.45 ± 0.15 b	33.5 ± 0.14 b	43.3 ± 0.27 a	4.23 ± 0.26 a	−8.97	3.34	22.61	
**Starch source**	PF—Tsai-ou lotus rhizome starch	N/A	N/A	27.74 ± 0.15 f	N/A	N/A	20.02 ± 0.86 e	N/A	N/A	−27.83	Yeh et al. 2021 [56]
	WS—Shih-lian lotus rhizome starch	N/A	N/A	35.39 ±0.56 d	N/A	N/A	16.52 ± 1.43 c	N/A	N/A	−53.32	
**Starch source**	Pea starch	19.2 ± 1.0 d	40.3 ± 1.6 b	40.5 ± 1.5 a	21.2 ± 0.5 c	43.2 ± 0.9 a	35.6 ± 0.8 b	10.42	7.20	−12.10	Chung et al. 2010 [57]
	Lentil starch	14.8 ± 0.9 d	41.5 ± 1.2 d	43.7 ± 1.3 a	18.8 ± 0.4 c	44 ± 0.7 c	37.2 ± 0.4 b	27.03	6.02	−14.87	
	Navy bean starch	8.2 ± 0.7 a	32.3 ± 0.6 c	59.4 ± 0.3 a	7.4 ± 1.0 ab	38.8 ± 0.8 b	53.9 ± 0.3 c	−9.76	20.12	−9.26	
**Starch source & Process temperature**	Corn Starch	29.7 ± 1.9	65.7 ± 1.3	4.6 ± 1.8	43.4 ± 0.6	48.6 ± 2.2	8.0 ± 1.7	46.13	−26.03	73.91	Chung et al. 2009 [41]
		29.7 ± 1.9	65.7 ± 1.3	4.6 ± 1.8	59.9 ± 1.7	31.4 ± 0.3	8.7 ± 1.5	101.68	−52.21	89.13	
	Pea starch	27.0 ± 2.5	62.9 ± 0.7	10 ± 2.4	29.3 ± 1.0	59.8 ± 1.3	10.9 ± 1.3	8.52	−4.93	9.00	
		27.0 ± 2.5	62.9 ± 0.7	10 ± 2.4	53.4 ± 1.1	34.4 ± 0.5	11.2 ± 1.3	97.78	−45.31	12.00	
	Lentil starch	20.8 ± 1.8	70.1 ± 2.2	9.1 ± 1.2	30.4 ± 1.6	60.4 ± 1.8	9.2 ± 2.1	46.15	−13.84	1.10	
		20.8 ± 1.8	70.1 ± 2.2	9.1 ± 1.2	57.8 ± 1.7	30.8 ± 1.5	11.4 ± 0.5	177.88	−56.06	25.27	
**Starch source**	Grass pea starch Derek	19.6 ± 0.4 ab	39.1 ± 1.5 i	41.3 ± 1.1 k	19.9 ± 0.6 b	42.3 ± 0.3 j	37.8 ± 0.9 j	1.53	8.18	−8.47	Piecyk et al. 2018 [42]
	Grass pea starch Krab	17.8 ± 0.8 ab	33.6 ± 1.4 h	48.6 ± 1.5 l	16.9 ± 0.9 a	40.8 ± 0.9 ij	42.3 ± 1.7 k	−5.06	21.43	−12.96	
**Duration**	Highland barley starch	91.19 ± 1.30 a	5.54 ± 0.41 e	3.27 ± 0.50 c	90.65 ± 3.47 a	5.23 ± 1.11 e	4.12 ± 0.13 c	−0.65	−5.60	25.99	Gu et al. 2025 [58]
		91.19 ± 1.30 a	5.54 ± 0.41 e	3.27 ± 0.50 c	87.52 ± 2.33 c	8.94 ± 0.79 a	3.54 ± 0.44 c	−4.05	61.37	8.26	
**Starch source &** **Moisture**	Yam *Dioscorea alata* starch	61.11 ± 0.17 b	15.68 ± 0.88 f	22.32 ± 0.19 g	45.29 ± 0.35 e	19.44 ± 0.26 d	34.67 ± 0.19 c	−25.89	23.98	55.33	Bora et al. 2023 [59]
		61.11 ± 0.17 b	15.68 ± 0.88 f	22.32 ± 0.19 g	37.83 ± 0.18 g	21.26 ± 0.16 b	40.63 ± 0.11 a	−38.10	35.59	82.03	
	Yam *Dioscorea esculenta* starch	70.22 ± 0.17 a	13.35 ± 0.55 g	15.51 ± 0.31 h	41.31 ± 0.56 f	20.72 ± 0.21 bc	36.72 ± 0.57 b	−41.17	55.21	136.75	
		70.22 ± 0.17 a	13.35 ± 0.55 g	15.51 ± 0.31 h	34.23 ± 0.21 h	24.18 ± 0.35 a	41.01 ± 0.12 a	−51.25	81.12	164.41	
**Starch source**	Rice starch	86.26 ± 0.13 a	10.48 ± 0.76 l	3.26 ± 0.83 h	52.14 ± 0.04 g	42.31 ± 0.93 h	5.55 ± 0.25 f	−39.55	303.72	70.25	Rosa-Millán 2017 [60]
	Maize starch	80.26 ± 0.28 c	15.38 ± 0.74 k	4.36 ± 0.47 g	55.26 ± 0.61 f	37.11 ± 0.05 j	7.63 ± 0.18 e	−31.15	141.29	75.00	
	Potato starch	86.99 ± 0.40 b	10.36 ± 0.35 k	2.65 ± 0.26 i	60.33 ± 0.81 d	33.22 ± 0.74 j	6.45 ± 0.55 e	−30.65	220.66	143.40	
**Duration**	Canna starch	51.97 ± 0.98	21.29 ± 0.15	26.74 ± 1.86	59.58 ± 3.55	6.16 ± 0.46	34.26 ± 3.08	14.64	−71.07	28.12	Lan et al. 2016 [61]
		51.97 ± 0.98	21.29 ± 0.15	26.74 ± 1.86	59.88 ± 1.23	9.68 ± 0.67	30.44 ± 1.9	15.22	−54.53	13.84	
		51.97 ± 0.98	21.29 ± 0.15	26.74 ± 1.86	64.11 ± 1.92	12.23 ± 0.18	23.66 ± 1.6	23.36	−42.56	−11.52	
		51.97 ± 0.98	21.29 ± 0.15	26.74 ± 1.86	65.61 ± 0.47	12.73 ± 0.72	21.66 ± 0.26	26.25	−40.21	−19.00	
	Waxy rice starch	32.4 ± 1.4 c	45.5 ± 2.0 bc	22.1 ± 1.7 a	38.7 ± 0.3 b	43.6 ± 1.3 bc	17.7 ± 1.2 ab	19.44	−4.18	−19.91	Zeng et al. 2015 [62]
	Kithul (*Caryota* *urens*) starch	19.89	30.99	49.26	17.58	29.83	53.42	−11.63	−3.73	8.45	Sudheesh et al. 2020 [63] **
	Common buckwheat starch	45.49	51.86	2.65	38.81	57.53	3.66	−14.68	10.94	38.00	Liu et al. 2015 [64] *^,^**
	Kithul palm starch	19.82 ± 0.25 e	31.16 ± 0.11 c	49.02 ± 0.13 a	10.88 ± 0.18 a	31.01 ± 0.24 c	58.11 ± 0.38 b	−45.11	−0.48	18.54	Sudheesh et al. 2019 [65]
	Water caltrop starch	5.69 ± 0.16 b	49.31 ± 0.48	37.18 ± 0.55 c	7.71 ± 0.12 e	46.67 ± 1.51	37.74 ± 1.30 c	35.50	−5.35	1.51	Liu et al. 2021 [66]
	Chinese yam (*Dioscorea opposita* Thunb.) starch	69.99 ± 0.07 a	14.37 ± 0.17 f	15.64 ± 0.23 f	65.43 ± 0.19 c	16.69 ± 0.23 d	17.88 ± 0.08 d	−6.52	16.14	14.32	Yu et al. 2021 [67]
	Native quinoa starch	38.21	42.17	14.19	33.99	43.22	18.68	−11.05	2.50	31.60	Liu et al. 2026 [40] **
	Corn starch	14.8 ± 0.1 a	57.6 ± 1.5 a	27.6 ± 1.4 a	19.7 ± 0.5 a	50.4 ± 1.4 a	29.9 ± 1.0 a	33.11	−12.50	8.33	Liang et al. 2020 [68]
	Bitter vetch (*Vicia ervilia*) starch	34.54 ± 0.16 e	18.03 ± 0.25 c	47.43 ± 0.21 a	44.91 ± 0.14 c	17.92 ± 0.24 c	37.17 ± 0.12 c	30.02	−0.61	−21.63	Tarahi et al. 2025 [69]
	White sorghum starch	N/A	N/A	43.70 a	N/A	N/A	55.20 b	N/A	N/A	26.32	Giuberti et al. 2019 [70]

* The values were extracted from a bar chart. The RDS, SDS, and RS values did not sum to 100%, so a normalization calculation was performed to obtain a total of 100%. ** The values were extracted from a bar chart. Percentage changes are calculated relative to each study’s native-starch control and should not be interpreted as direct rankings of process efficiency, due to differences in RS assays, drying procedures, moisture basis, and starch sources across studies (see Section 3.2). Letter denote statistically different values (*p* < 0.05). Color code red indicates processing compromises digestibility while green color indicates a positive impact.

**Table 2:** Alteration percentage after applying the HMT process.

Comparison Parameters	Starch	Native Starch			Modified Starch			% Alteration (vs. Native Baseline)			Ref
		RDS ± SD	SDS ± SD	RS ± SD	RDS ± SD	SDS ± SD	RS ± SD	RDS	SDS	RS	
**Moisture**	Rice starch	93.9 ± 1.5 a	3.5 ± 0.8 c	2.6 ± 0.5 d	84 ± 2.1 b	8.3 ± 0.7 b	7.7 ± 0.8 c	−10.54	137.14	196.15	Wang et al. 2018 [46]
		93.9 ±1.5 a	3.5 ± 0.8 c	2.6 ± 0.5 d	75.2 ± 2.3 c	11 ± 0.7 a	13.8 ± 1.0 b	−19.91	214.29	430.77	
		93.9 ±1.5 a	3.5 ± 0.8 c	2.6 ± 0.5 d	75.0 ± 2.1 c	10.5 ± 1.6 a	14.5 ± 1.9 b	−20.13	200.00	457.69	
**Moisture**	Red Adzuki bean starch	86 ± 3.5 a	2.3 ± 2.1 e	11.7 ± 2.5 a	81.1 ± 2.6 b	9.7 ± 2.3 d	9.2 ± 2.7 b	−5.70	321.74	−21.37	Wang et al. 2017 [71]
		86 ± 3.5 a	2.3 ± 2.1 e	11.7 ± 2.5 a	80.2 ± 3.4 b	11.1± 1.8 c	8.7 ± 2.3 b	−6.74	382.61	−25.64	
		86 ± 3.5 a	2.3 ± 2.1 e	11.7 ± 2.5 a	78.1 ± 3.2 bc	14.2 ± 1.5 b	7.7 ± 1.6 bc	−9.19	517.39	−34.19	
		86 ± 3.5 a	2.3 ± 2.1 e	11.7 ± 2.5 a	74.3 ± 2.8 d	15.8 ± 1.3 b	9.9 ± 1.7 b	−13.60	586.96	−15.38	
		86 ± 3.5 a	2.3 ± 2.1 e	11.7 ± 2.5 a	71.3 ± 3.0 e	20.9 ± 2.2 a	7.8 ± 2.4 bc	−17.09	808.70	−33.33	
**Starch source**	Rice starch	86.26 ± 0.13 a	10.48 ± 0.76 l	3.26 ± 0.83 h	35.36 ± 0.69 k	56.93 ± 0.01 c	7.71 ± 0.20 e	−59.01	443.23	136.50	Rosa-Millán 2017 [60]
	Maize starch	80.26 ± 0.28 c	15.38 ± 0.74 k	4.36 ± 0.47 g	52.17 ± 0.95 g	42.3 ± 0.82 h	5.53 ± 0.04 f	−35.00	175.03	26.83	
	Potato starch	86.99 ± 0.40 b	10.36 ± 0.35 k	2.65 ± 0.26 i	55.63 ± 0.52 f	35.71 ± 0.43 j	8.66 ± 0.90 d	−36.05	244.69	226.79	
**Temperature**	Sweet potato starch	53.82 ± 0.13 a	28.27 ± 2.24 e	17.92 ± 2.37 d	36.88 ± 2.62 d	39.59 ± 2.97 a	23.54 ± 0.35 c	−31.48	40.04	31.36	Sun et al. 2023 [47]
		53.82 ± 0.13 a	28.27 ± 2.24 e	17.92 ± 2.37 d	37.13 ± 0.18 c	38.11 ± 0.17 b	24.77 ± 0.35 a	−31.01	34.81	38.23	
		53.82 ± 0.13 a	28.27 ± 2.24 e	17.92 ± 2.37 d	37.99 ± 0.35 c	37.86 ± 0.52 c	24.15 ± 0.17 b	−29.41	33.92	34.77	
		53.82 ± 0.13 a	28.27 ± 2.24 e	17.92 ± 2.37 d	39.72 ± 0.70 b	35.77 ± 0.35 d	24.52 ±1.05 ab	−26.20	26.53	36.83	
**Moisture**	Jackfruit seed starch	15.96 d	58.82 d	25.22 a	11.19 b	46.04 b	42.77 c	−29.89	−21.73	69.59	Vu et al. 2024 [72]
		15.96 d	58.82 d	25.22 a	10.36 a	36.02 a	53.62 d	−35.09	−38.76	112.61	
		15.96 d	58.82 d	25.22 a	12.82 c	46.63 c	40.55 b	−19.67	−20.72	60.79	
**Moisture & Starch source**	Regular maize starch (RMS)	91.7 ±1.7 a	6.2 ± 1.3 e	2.1 ± 0.3 f	82.9 ± 2.3 b	12.9 ± 1.5 c	4.2 ± 1.3 f	−9.60	108.06	100.00	Wang et al. 2016 [73]
		91.7 ± 1.7 a	6.2 ± 1.3 e	2.1 ± 0.3 f	76.8 ± 1.8 c	9.1 ± 1.5 d	14.1 ± 2.1 c	−16.25	46.77	571.43	
		91.7 ± 1.7 a	6.2 ± 1.3 e	2.1 ± 0.3 f	69.4 ± 1.9 d	18.9 ± 2.1 a	11.7 ± 1.5 d	−24.32	204.84	457.14	
	Gelose 50 high-amylose maize starch (G50)	86 ± 2.2 b	7.4 ± 1.1 e	6.6 ± 1.4 e	78.6 ± 1.7 c	10.2 ± 1.8 d	11.2 ± 1.3 d	−8.60	37.84	69.70	
		86 ± 2.2 b	7.4 ± 1.1 e	6.6 ± 1.4 e	63.8 ± 2.1 e	15.8 ± 1.6 bc	20.4 ± 2.4 b	−25.81	113.51	209.09	
		86 ± 2.2 b	7.4 ± 1.1 e	6.6 ± 1.4 e	51.7 ± 2.2 f	18.8 ± 2.1 a	29.5 ± 2.4 a	−39.88	154.05	346.97	
**Temperature & Starch source**	Wrinkled pea starch (MPG87)	70.3 ± 0.7 c	2.1 ± 0.4 abc	21.4 ± 0.6 d	63.6 ± 0.4 a	2.6±0.1 bcd	27.5 ± 0.5 ef	−9.53	23.81	28.50	Cheng et al. 2024 [35]
		70.3 ± 0.7 c	2.1 ± 0.4 abc	21.4 ± 0.6 d	62.9 ± 0.4 a	3.6±0.8 cd	29.9 ± 0.1 f	−10.53	71.43	39.72	
	Wrinkled pea starch (CDC 4140-4)	73.5 ± 0.5 d	2.8 ± 0.5 bcd	18.2 ± 0.7 c	64.9 ± 1.2 a	2.5±1.5 abcd	25.8 ± 1.7 e	−11.70	−10.71	41.76	
		73.5 ± 0.5 d	2.8 ± 0.5 bcd	18.2 ± 0.7 c	67.5 ± 0.8 b	4.2±0.6 d	22.7 ± 0.1 d	−8.16	50.00	24.73	
	Round pea starch (Terese)	86 ± 0.1 gh	1 ± 0.6 ab	6.2 ± 0.5 a	83.1 ± 1.6 f	1.1±0.6 ab	10.8 ± 1.3 b	−3.37	10.00	74.19	
		86 ± 0.1 gh	1 ± 0.6 ab	6.2 ± 0.5 a	80.9 ± 1.2 e	3.6±1 cd	10.6 ± 1.7 b	−5.93	260.00	70.97	
	Round pea starch (CDC Bronco)	87.4 ± 0.7 h	0.9 ± 0.2 ab	5.8 ± 0.7 a	85.1 ± 1 fg	0.4±0.4 a	9.8 ± 1.4 b	−2.63	−55.56	68.97	
		87.4 ± 0.7 h	0.9 ± 0.2 ab	5.8 ± 0.7 a	83.3 ± 0.8 f	1.8±0.5 ab	11 ± 0.8 b	−4.69	100.00	89.66	
**Moisture**	Water caltrop starch	5.69 ± 0.16 b	49.31 ± 0.48	37.18 ± 0.55 c	4.03 ± 0.09 a	49.08± 3.11	40.19 ± 0.63 d	−29.17	−0.47	8.10	Liu et al. 2021 [66]
		5.69 ± 0.16 b	49.31 ± 0.48	37.18 ± 0.55 c	8.4 ± 0.12 f	49.49 ±1.70	32.44 ± 0.05 b	47.63	0.37	−12.75	
		5.69 ± 0.16 b	49.31 ± 0.48	37.18 ± 0.55 c	26.29 ± 0.05 g	48.46 ±2.26	17.01 ± 0.08 a	362.04	−1.72	−54.25	
**Moisture & Starch source**	PF—Tsai-ou lotus rhizome starch	N/A	N/A	27.74 ± 0.15 f	N/A	N/A	8.29± 0.14 d	N/A	N/A	−70.12	Yeh et al. 2021 [56]
		N/A	N/A	27.74 ± 0.15 f	N/A	N/A	5.4± 0.11 b	N/A	N/A	−80.53	
		N/A	N/A	27.74 ± 0.15 f	N/A	N/A	2.69 ± 0.04 a	N/A	N/A	−90.30	
	WS—Shih-lian lotus rhizome starch	N/A	N/A	35.39 ± 0.56 d	N/A	N/A	5.57 ± 0.06 b	N/A	N/A	−84.26	
		N/A	N/A	35.39 ± 0.56 d	N/A	N/A	3.35 ± 0.05 a	N/A	N/A	−90.53	
		N/A	N/A	35.39 ± 0.56 d	N/A	N/A	2.68 ± 0.10 a	N/A	N/A	−92.43	
**Moisture**	Common buckwheat starch	45.49	51.86	2.65	41.42	55.83	2.76	−8.96	7.65	4.12	Liu et al. 2015 [64] *^,^**
		45.49	51.86	2.65	36.24	60.62	3.14	−20.33	16.88	18.68	
		45.49	51.86	2.65	35.27	61.15	3.58	−22.47	17.91	35.26	
		45.49	51.86	2.65	31.50	64.53	3.98	−30.76	24.42	50.10	
**Starch source**	Pea	19.2 ± 1.0 d	40.3 ± 1.6 b	40.5 ± 1.5 a	25.4 ± 0.8 b	45.3 ± 2.2 a	29.2 ± 1.7 d	32.29	12.41	−27.90	Chung et al. 2010 [57]
	Lentil	14.8 ± 0.9 d	41.5 ± 1.2 d	43.7 ± 1.3 a	21.7 ± 0.3 b	49.2 ± 1.3 a	29 ± 1.0 d	46.62	18.55	−33.64	
	Navy bean	8.2 ± 0.7 a	32.3 ± 0.6 c	59.4 ± 0.3 a	7.6 ± 0.2 ab	42.5 ± 1.2 a	49.9 ± 1.2 d	−7.32	31.58	−15.99	
**Starch source**	Grass pea starch Derek	19.6 ± 0.4 ab	39.1 ± 1.5 i	41.3 ± 1.1 k	18.6 ± 0.7 ab	49.5 ± 0.8 k	31.9 ± 1.5 h	−5.10	26.60	−22.76	Piecyk et al. 2018 [42]
	Grass pea starch Krab	17.8 ± 0.8 ab	33.6 ± 1.4 h	48.6 ± 1.5 l	16.7 ± 1.2 a	48.7 ± 0.7 k	34.6 ± 1.0 i	−6.18	44.94	−28.81	
**Temperature & Starch source**	Corn	29.7 ± 1.9	65.7 ± 1.3	4.6 ± 1.8	65.2 ± 1.3	24.2 ± 1.4	10.5 ±1.0	119.53	−63.17	128.26	Chung et al. 2009 [41]
		29.7 ± 1.9	65.7 ± 1.3	4.6 ± 1.8	70.4 ± 0.4	17.3 ± 2.2	12.3 ±1.9	137.04	−73.67	167.39	
	Pea	27.0 ± 2.5	62.9 ± 0.7	10 ± 2.4	60.8 ± 0.8	25.9 ± 2.0	13.3 ±1.5	125.19	−58.82	33.00	
		27.0 ± 2.5	62.9 ± 0.7	10 ± 2.4	71.7 ± 0.7	13.8 ± 1.0	14.5 ±1.2	165.56	−78.06	45.00	
	Lentil	20.8 ± 1.8	70.1 ± 2.2	9.1 ± 1.2	56.4 ± 2.0	30.4 ± 2.2	13.2 ±0.5	171.15	−56.63	45.05	
		20.8 ± 1.8	70.1 ± 2.2	9.1 ± 1.2	61.7 ± 1.6	23.6 ± 3.0	14.7 ±2.0	196.63	−66.33	61.54	
**Moisture**	Highland barley starch	91.19 ± 1.30 a	5.54 ± 0.41 e	3.27 ± 0.50 c	81.5 ± 1.56 d	8.73 ± 0.52 b	9.77 ± 0.77 b	−10.63	57.58	198.78	Gu et al. 2025 [58]
		91.19 ± 1.30 a	5.54 ± 0.41 e	3.27 ± 0.50 c	82.2 ± 1.34 d	5.25 ± 0.35 e	12.5 ± 0.60 a	−9.86	−5.23	282.26	
		91.19 ± 1.30 a	5.54 ± 0.41 e	3.27 ± 0.50 c	84.5 ± 1.60 d	6.11 ± 0.30 d	9.35 ± 0.58 b	−7.34	10.29	185.93	
**Moisture & Starch source**	Yam *Dioscorea alata* starch	61.11 ± 0.17 b	15.68 ± 0.88 f	22.32 ± 0.19 g	55.71 ± 0.39 c	17.14 ± 0.44 e	26.13 ± 0.32 e	−8.84	9.31	17.07	Bora et al. 2023 [59]
		61.11 ± 0.17 b	15.68 ± 0.88 f	22.32 ± 0.19 g	46.37 ± 0.58 d	21.25 ± 0.18 b	31.51 ± 0.19 d	−24.12	35.52	41.17	
	Yam *Dioscorea esculenta* starch	70.22 ± 0.17 a	13.35 ± 0.55 g	15.51 ± 0.31 h	61.13 ± 0.27 b	16.25 ± 0.46 ef	21.38 ± 0.58 g	−12.95	21.72	37.85	
		70.22 ± 0.17 a	13.35 ± 0.55 g	15.51 ± 0.31 h	56.25 ± 0.11 c	19.53 ± 0.14 cd	23.84 ± 0.23 f	−19.89	46.29	53.71	
	Corn starch	14.8 ± 0.1 a	57.6 ± 1.5 a	27.6 ± 1.4 a	21.8 ± 1.5 a	40.8 ± 0.3 a	37.4 ± 1.2 a	47.30	−29.17	35.51	Liang et al. 2020 [68]
	Banana starch	32.64	11.57	55.79	62.02	20.18	17.80	90.00	74.36	−68.09	Sun et al. 2025 [74] **
	Rice starch	30.6 ± 1.5 b	56.4 ± 1.1 a	13 ± 0.5 b	52.5 ± 2.6 a	31.6 ± 1.3 b	15.9 ± 2.7 a	71.57	−43.97	22.31	Xie et al. 2020 [75]
	Talipot starch	29.44 ± 0.05 f	33.85 ± 0.11 d	36.71 ± 0.02 b	27.71 ± 0.16 e	33.31 ± 0.02 c	38.98 ± 0.18 c	−5.88	−1.60	6.18	Aaliya et al. 2021 [76]
	Proso millet starch	N/A	N/A	11.50± 0.3 c	N/A	N/A	12.30 ± 0.7 c	N/A	N/A	6.96	Zheng et al. 2020 [51]
	Kithul (*Caryota* *urens*) starch	19.89	30.99	49.26	15.49	27.75	57.35	−22.09	−10.45	16.43	Sudheesh et al. 2020 [63]
	White sorghum starch	N/A	N/A	43.70 a	N/A	N/A	55.70 b	N/A	N/A	27.46	Giuberti et al. 2019 [70]
	Waxy rice starch	32.4 ± 1.4 c	45.5 ± 2.0 bc	22.1 ± 1.7 a	27.3 ± 0.8 d	54.6 ± 1.8 a	18.1 ± 1.4 ab	−15.74	20.00	−18.10	Zeng et al. 2015 [62]
	Yam starch	69.99 ± 0.07 a	14.37 ± 0.17 f	15.64 ± 0.23 f	62.04 ± 0.10 e	17.89 ± 0.04 b	20.07 ± 0.08 b	−11.36	24.50	28.32	Yu et al. 2021 [67]
	Bitter vetch (*Vicia ervilia*) starch	34.54 ± 0.16 e	18.03 ± 0.25 c	47.43 ± 0.21 a	45.48 ± 0.16 b	20.78 ± 0.16 b	33.74 ± 0.09 e	31.67	15.25	−28.86	Tarahi et al. 2025 [69]

* The values were extracted from a bar chart. The RDS, SDS, and RS values did not sum to 100%, so a normalization calculation was performed to obtain a total of 100%. ** The values were extracted from a bar chart. Percentage changes are calculated relative to each study’s native-starch control and should not be interpreted as direct rankings of process efficiency, due to differences in RS assays, drying procedures, moisture basis, and starch sources across studies (see Section 3.2). Color code red indicates processing compromises digestibility while green color indicates a positive impact.

**Table 3:** Alteration percentage after applying the autoclaving process.

Comparison Parameters	Starch	Native Starch			Modified Starch			% Alteration (vs. Native Baseline)			Ref
		RDS ± SD	SDS ± SD	RS ± SD	RDS ± SD	SDS ± SD	RS ± SD	RDS	SDS	RS	
**Temperature &** **Starch source**	Normal corn starch (NS)	N/A	N/A	2.10 ±1.0 c	N/A	N/A	3.30 ± 0.0 b	N/A	N/A	57.14	Soler et al. 2020 [53]
		N/A	N/A	2.10 ± 1.0 c	N/A	N/A	5.30 ± 0.1 a	N/A	N/A	152.38	
		N/A	N/A	2.10 ± 1.0 c	N/A	N/A	5.50 ± 0.2 a	N/A	N/A	161.90	
	Hylon VII (HS)	N/A	N/A	12.70 ± 1.6 d	N/A	N/A	20.20 ± 0.3 c	N/A	N/A	59.06	
		N/A	N/A	12.70 ± 1.6 d	N/A	N/A	30.20 ± 1.7 a	N/A	N/A	137.80	
		N/A	N/A	12.70 ± 1.6 d	N/A	N/A	27.10 ± 1.0 b	N/A	N/A	113.39	
**Temperature & Retrogradation**	Cilacap breadfruit starch	N/A	N/A	10.98 ± 0.07 f	N/A	N/A	11.03 ± 0.06 f	N/A	N/A	0.46	Rahayu et al. 2023 [52]
		N/A	N/A	10.98 ± 0.07 f	N/A	N/A	12.92 ± 0.05 d	N/A	N/A	17.67	
		N/A	N/A	10.98 ± 0.07 f	N/A	N/A	18.51 ± 0.11 b	N/A	N/A	68.58	
		N/A	N/A	10.98 ± 0.07 f	N/A	N/A	11.88 ± 0.17 e	N/A	N/A	8.20	
		N/A	N/A	10.98 ± 0.07 f	N/A	N/A	14.14 ± 0.19 c	N/A	N/A	28.78	
		N/A	N/A	10.98 ± 0.07 f	N/A	N/A	19.41 ± 0.05 a	N/A	N/A	76.78	
**Cycles**	Waxy corn starch	86.00 ± 0.2 a	4.00 ± 3.2 a	10.10 ± 3.1 a	83.30 ± 3.0 ab	3.50 ± 1.8 a	13.20 ± 1.3 a	−3.14	−12.50	30.69	Sung et al. 2020 [77]
		86.00 ± 0.2 a	4.00 ± 3.2 a	10.10 ± 3.1 a	82.90 ± 2.4 ab	4.10 ± 2.1 a	13.00 ± 1.9 a	−3.60	2.50	28.71	
		86.00 ± 0.2 a	4.00 ± 3.2 a	10.10 ± 3.1 a	81.40 ± 2.3 b	5.30 ± 2.3 a	13.40 ± 1.3 a	−5.35	32.50	32.67	
**Cycles**	Cowpea starch	N/A	N/A	32.14 ± 1.33 b	N/A	N/A	41.26 ± 0.81 a	N/A	N/A	28.38	Ratnaningsih et al. 2020 [78]
		N/A	N/A	32.14 ± 1.33 b	N/A	N/A	31.83 ± 0.53 b	N/A	N/A	−0.96	
		N/A	N/A	32.14 ± 1.33 b	N/A	N/A	33.95 ± 1.82 b	N/A	N/A	5.63	
**Cycles & Starch sources**	Non-waxy rice KDML105	N/A	N/A	0.13 ± 0.01 de	N/A	N/A	0.65 ± 0.02 c	N/A	N/A	400.00	
		N/A	N/A	0.13 ± 0.01 de	N/A	N/A	1.27 ± 0.06 b	N/A	N/A	876.92	Sangwongchai et al. 2024 [32]
		N/A	N/A	0.13 ± 0.01 de	N/A	N/A	2.59 ± 0.02 a	N/A	N/A	1892.31	
	Waxy rice RD6	N/A	N/A	0.17 ± 0.01 d	N/A	N/A	0.10 ± 0.01 def	N/A	N/A	−41.18	
		N/A	N/A	0.17± 0.01 d	N/A	N/A	0.06 ± 0.00 ef	N/A	N/A	−64.71	
		N/A	N/A	0.17± 0.01 d	N/A	N/A	0.02 ± 0.00 f	N/A	N/A	−88.24	
**Starch sources**	Rice starch: SR-1	N/A	N/A	4.42 ± 0.88 a	N/A	N/A	30.31 ± 0.79 e	N/A	N/A	585.75	Ashwar et al. 2016 [79]
	Rice starch: SR-2	N/A	N/A	8.26 ± 0.86 c	N/A	N/A	35.80 ± 0.90 g	N/A	N/A	333.41	
	Rice starch: Pusa sugandh	N/A	N/A	5.91 ± 0.61 b	N/A	N/A	32.56 ± 0.63 f	N/A	N/A	450.93	
	Rice starch: Jhelum	N/A	N/A	10.94 ± 0.59 d	N/A	N/A	38.65 ± 0.82 h	N/A	N/A	253.29	
**Starch sources**	NCS–Normal amylose starch	53.92 ± 3.05 a	26.15 ± 2.69 a	19.93 ± 1.86 f	46.88 ± 11.77 ab	12.10 ± 7.50 b	41.01 ± 0.44 d	−13.06	−53.73	105.77	Li et al. 2025 [29]
	HACS-55 high-amylose corn	48.07 ± 1.79 ab	16.44 ± 0.98 b	35.50 ± 2.21 e	40.93 ± 2.10 bc	13.09 ± 0.84 b	45.98 ± 1.28 c	−14.85	−20.38	29.52	
	HACS-70 high-amylose corn starch	34.90 ± 3.98 cd	12.37 ± 4.49 b	52.73 ± 0.76 b	30.81 ± 1.39 d	12.03 ± 1.14 b	57.16 ± 0.37 a	−11.72	−2.75	8.40	
**Duration**	Cassava starch	N/A	N/A	0.44	N/A	N/A	11.40	N/A	N/A	2490.91	Onyango et al. 2006 [27] **
		N/A	N/A	0.44	N/A	N/A	11.91	N/A	N/A	2606.76	
		N/A	N/A	0.44	N/A	N/A	10.66	N/A	N/A	2322.39	
		N/A	N/A	0.44	N/A	N/A	11.21	N/A	N/A	2448.78	
		N/A	N/A	0.44	N/A	N/A	11.77	N/A	N/A	2575.17	
**Retrogradation**		N/A	N/A	0.44	N/A	N/A	3.02	N/A	N/A	586.36	
		N/A	N/A	0.44	N/A	N/A	3.79	N/A	N/A	761.36	
		N/A	N/A	0.44	N/A	N/A	4.78	N/A	N/A	986.36	
		N/A	N/A	0.44	N/A	N/A	5.23	N/A	N/A	1088.64	
		N/A	N/A	0.44	N/A	N/A	5.97	N/A	N/A	1256.82	
		N/A	N/A	0.44	N/A	N/A	6.50	N/A	N/A	1377.27	
		N/A	N/A	0.44	N/A	N/A	4.80	N/A	N/A	990.91	
		N/A	N/A	0.44	N/A	N/A	5.93	N/A	N/A	1247.73	
		N/A	N/A	0.44	N/A	N/A	5.61	N/A	N/A	1175.00	
		N/A	N/A	0.44	N/A	N/A	3.49	N/A	N/A	693.18	
		N/A	N/A	0.44	N/A	N/A	4.96	N/A	N/A	1027.27	
		N/A	N/A	0.44	N/A	N/A	6.04	N/A	N/A	1272.73	
		N/A	N/A	0.44	N/A	N/A	1.74	N/A	N/A	295.45	
		N/A	N/A	0.44	N/A	N/A	9.47	N/A	N/A	2052.27	
		N/A	N/A	0.44	N/A	N/A	9.68	N/A	N/A	2100.00	
**Moisture & Retrogradation**	Culinary banana starch	N/A	N/A	18.88 ± 0.05	N/A	N/A	9.45 ± 0.75 a	N/A	N/A	−49.95	Khawas et al. 2017 [33]
		N/A	N/A	18.88 ± 0.05	N/A	N/A	10.23 ± 0.06 b	N/A	N/A	−45.82	
		N/A	N/A	18.88 ± 0.05	N/A	N/A	12.30 ± 0.09 d	N/A	N/A	−34.85	
		N/A	N/A	18.88 ± 0.05	N/A	N/A	12.30 ± 0.05 a	N/A	N/A	−34.85	
		N/A	N/A	18.88 ± 0.05	N/A	N/A	15.43 ± 0.07 b	N/A	N/A	−18.27	
		N/A	N/A	18.88 ± 0.05	N/A	N/A	11.98 ± 0.14 c	N/A	N/A	−36.55	
		N/A	N/A	18.88 ± 0.05	N/A	N/A	14.00 ± 0.01 e	N/A	N/A	−25.85	
		N/A	N/A	18.88 ± 0.05	N/A	N/A	23.31 ± 0.67 f	N/A	N/A	23.46	
		N/A	N/A	18.88 ± 0.05	N/A	N/A	23.79 ± 0.60 e	N/A	N/A	26.01	
		N/A	N/A	18.88 ± 0.05	N/A	N/A	26.42 ± 0.12 d	N/A	N/A	39.94	
**Cycles & Retrogradation**	Khai Mod Rin rice starch	71.67 ± 0.16 b	13.96 ± 0.33 a	14.37 ± 0.10 g	69.03 ± 0.18 c	12.72 ± 0.28 a	18.26 ± 0.08 f	−3.68	−8.88	27.07	Junden et al. 2025 [54]
		71.67 ± 0.16 b	13.96 ± 0.33 a	14.37 ± 0.10 g	63.31 ± 0.44 d	8.60 ± 0.57 b	28.09 ± 0.13 e	−11.66	−38.40	95.48	
		71.67 ± 0.16 b	13.96 ± 0.33 a	14.37 ± 0.10 g	62.89 ± 0.33 d	8.73 ± 0.14 b	28.38 ± 0.48 e	−12.25	−37.46	97.49	
		71.67 ± 0.16 b	13.96 ± 0.33 a	14.37 ± 0.10 g	62.53 ± 0.32 d	8.45 ± 0.07 bc	29.02 ± 0.03 e	−12.75	−39.47	101.95	
		71.67 ± 0.16 b	13.96 ± 0.33 a	14.37 ± 0.10 g	55.52 ± 0.42 f	7.56 ± 0.56 bcd	36.92 ± 0.12 e	−22.53	−45.85	156.92	
		71.67 ± 0.16 b	13.96 ± 0.33 a	14.37 ± 0.10 g	43.32 ± 0.13 h	6.98 ± 0.12 cde	49.71 ± 0.41 a	−39.56	−50.00	245.93	
		71.67 ± 0.16 b	13.96 ± 0.33 a	14.37 ± 0.10 g	59.55 ± 0.79 e	8.28 ± 0.13 bc	32.16 ± 0.06 d	−16.91	−40.69	123.80	
		71.67 ± 0.16 b	13.96 ± 0.33 a	14.37 ± 0.10 g	57.35 ± 0.49 f	5.80 ± 0.71 e	36.85 ± 0.19 e	−19.98	−58.45	156.44	
		71.67 ± 0.16 b	13.96 ± 0.33 a	14.37 ± 0.10 g	53.01 ± 0.83 g	6.41 ± 0.16 de	40.58 ± 0.38 b	−26.04	−54.08	182.39	
**Retro-gradation**	Potato starch	N/A	N/A	0.01	N/A	N/A	2.45	N/A	N/A	24400	Yaskin Harush et al. 2025 [80]
		N/A	N/A	0.01	N/A	N/A	2.86	N/A	N/A	28500	
**Drying & starch source**	Hylon V maize starch	N/A	N/A	43.00	N/A	N/A	27.30	N/A	N/A	−36.51	Ozturk et al. 2009 [30]
		N/A	N/A	43.00	N/A	N/A	17.70	N/A	N/A	−58.84	
	Hylon VII maize starch	N/A	N/A	53.00	N/A	N/A	29.10	N/A	N/A	−45.09	
		N/A	N/A	53.00	N/A	N/A	23.50	N/A	N/A	−55.66	
**Starch source**	Bean (*Phaseolus coccineus*) starch—Nata	25.60 ± 0.8 e	48.30 ± 0.8 a	20.40 ± 0.2 a	71.90 ± 2.5 c	9.20 ± 2.2 c	13.30 ± 0.8 e	180.86	−80.95	−34.80	Piecyk et al. 2013 [81]
	Bean (*Phaseolus coccineus*) starch—Karo	29.10 ± 0.4 d	44.50 ± 0.6 b	18.60 ± 0.4 b	76.50 ± 1.4 b	4.00 ± 0.2 d	11.70 ± 1.2 fg	162.89	−91.01	−37.10	
**Temperature** **& Retrogradation**	High-amylose corn starch Hylon VII (70%)	N/A	N/A	N/A	N/A	N/A	21.73	N/A	N/A	N/A	Dundar et al. 2013 [26] **
		N/A	N/A	N/A	N/A	N/A	22.91	N/A	N/A	N/A	
		N/A	N/A	N/A	N/A	N/A	26.82	N/A	N/A	N/A	
		N/A	N/A	N/A	N/A	N/A	24.94	N/A	N/A	N/A	
		N/A	N/A	N/A	N/A	N/A	26.36	N/A	N/A	N/A	
		N/A	N/A	N/A	N/A	N/A	30.41	N/A	N/A	N/A	
**Moisture**	Cassava root starch	N/A	N/A	N/A	N/A	N/A	8.70	N/A	N/A	N/A	Khurshida et al. 2022 [82]
		N/A	N/A	N/A	N/A	N/A	10.51	N/A	N/A	N/A	
	Purple sweet potato starch	N/A	N/A	1.41 ± 0.10 c	N/A	N/A	9.15 ± 0.07 b	N/A	N/A	535.42	Sun et al. 2022 [28]
	Banana Starch	N/A	N/A	1.51±0.1 a	N/A	N/A	16.02 ± 0.24 a	N/A	N/A	960.93	Aparicio-Saguilána et al. 2005 [83]
	Talipot starch	29.44 ± 0.05 f	33.85 ± 0.11 d	36.71 ± 0.02 b	22.54 ± 0.11 c	35.38 ± 0.02 e	42.08 ± 0.15 d	−23.44	4.52	14.63	Aaliya et al. 2021 [76]
	High-amylose maize (HAM, Hylon VII)	N/A	N/A	82.16±0.34	N/A	N/A	30.15±0.52 c	N/A	N/A	−63.30	Li et al. 2018 [84]
	Black Tartary buckwheat native starch	N/A	N/A	20.64	N/A	N/A	23.82	N/A	N/A	15.38	Zheng at al. 2023 [31] **
	Euryale ferox starch	N/A	N/A	10.13 ± 0.54 f	N/A	N/A	18.17 ± 0.01 d	N/A	N/A	79.37	Yang et al. 2021 [85]
	Purple sweet potato starch	13.42± 0.16 e	26.06 ± 0.01 a	60.51 ± 0.00 e	30.34 ± 0.05 a	13.58 ± 0.05 f	56.07 ± 0.00 g	126.08	−47.89	−7.34	Bodjrenou et al. 2022 [86]
	Proso millet starch	N/A	N/A	11.50± 0.3 c	N/A	N/A	15.50± 1.1 b	N/A	N/A	34.78	Zheng et al. 2020 [51]
	Kithul starch (*Caryota urens*)	19.89	30.99	49.26	21.51	30.53	48.33	8.14	−1.49	−1.88	Sudheesh et al. 2020 [63] **
	Lotus seed starch	2.12 ± 0.13 c	10.32 ± 2.26 b	87.55 ± 2.20 a	16.64 ± 1.03 a	25.11 ± 1.66 a	58.25 ± 1.43 c	684.91	143.31	−33.47	Wang et al. 2024 [87]
	Normal rice starch	91.50 ± 0.2 a	7.20 ± 0.2 d	1.10 ± 0.1 d	74.00 ± 0.4 b	17.10 ± 0.5 c	8.90 ± 0.4 c	−19.13	137.50	709.09	Li et al. 2020 [9]
	Buckwheat starch	33.38 ± 0.32 c	8.62 ± 0.69 d	58.00 ± 0.62 a	43.15 ± 0.35 a	18.67 ± 0.32 a	38.18 ± 0.34 d	29.27	116.59	-34.17	Tao et al. 2025 [88]
	White sorghum starch	N/A	N/A	43.70	N/A	N/A	56.90	N/A	N/A	30.21	Giuberti et al. 2019 [70]
	Rice starch	47.38 ± 4.84 ab	31.65 ± 2.71 ab	20.99 ± 2.14 d	49.76 ± 0.06 ab	22.96 ± 0.08 bc	27.28 ± 0.14 cf	5.02	−27.46	29.99	Frasson et al. 2024 [89]
	Banxia starch	6.99 ± 0.25 b	25.91 ± 0.08 b	63.07 ± 0.29 a	30.20 ± 0.47 a	37.71 ± 2.48 a	27.24 ± 2.43 b	332.05	45.54	−56.81	Li et al. 2021 [90]
	Chinese yam starch	26.52 ± 0.94 b	22.95 ± 1.4 b	50.53 ± 0.47 b	30.93 ± 0.46 a	27.28 ± 0.81 a	41.79 ± 0.43 d	16.63	18.87	−17.30	Xie et al. 2023 [91]
	Pea starch	N/A	N/A	0.065 ± 0.011 n	N/A	N/A	0.331 ± 0.043 k	N/A	N/A	409.23	Cui et al. 2021 [92]

** The values were extracted from a chart. *** It should be noted that articles including numerous parameter comparisons were still considered, even if they did not report the RDS, SDS, or RS values of native starch. The corresponding values in the tables were highlighted in red, as the authors considered it useful for understanding the effects of process parameters on the RDS/SDS/RS ratio. Percentage changes are calculated relative to each study’s native-starch control and should not be interpreted as direct rankings of process efficiency, due to differences in RS assays, drying procedures, moisture basis, and starch sources across studies (see Section 3.2). Color code red indicates processing compromises digestibility while green color indicates a positive impact.

**Table 4:** Comparison of ANN, HMT and autoclaving: recommended conditions, expected RS, mechanistic rationale, and process selection note specific to raw material.

Process	Recommended Conditions	Expected RRS/SDS/RDS Response	Rationale	Raw-Material/Process-Selection Note	Reference
Annealing **ANN**	Excess (>65% *w*/*w*) or intermediate (40–50% *w*/*w*) moisture; temperature above Tg and below To (typically To −10 to −15 °C); 24–72 h (up to several days)	Median RS +13.8% (range −53.3 to +164.4%, *n* = 47), mildest and most variable of the three processes; median RDS +0.4% (*n* = 44), SDS +4.6% (*n* = 44)	Below-gelatinization reorganization allows starch chains to anneal into a more ordered, less-disrupted crystalline arrangement without full granule breakdown, mildly increasing resistance to enzymatic hydrolysis	ANN is a hydrothermal method that is best characterized by modest and inconsistent effects across amylose classes. While it works reasonably on common and high-amylose starches (e.g., corn, pea, lentil), its effects are not reliable on waxy substrates where it can even reduce RS and increase RDS. Therefore, ANN is best used as a gentle, low-energy pre-treatment or when native granule integrity must be preserved, rather than as a primary RS-maximizing step.	[39,41,42,62]
High moisture and temperature **HMT**	Low moisture (<35% *w*/*w*); 80–140 °C (above Tg but below the effective gelatinization temperature of the starch under these limited-moisture conditions); several hours, single step	Median RS +34.8% (range −92.4 to +571.4%, n = 73); median RDS −9.9% (n = 65), SDS +24.5% (n = 65); most consistent RDS-lowering effect and increasing SDS.	Restricted-mobility, low-moisture heating promotes amylose–amylose interactions and partial crystallite disruption/reorganization, favoring a shift from RDS toward SDS.	HMT is effective on high-amylose starches (e.g., wrinkled pea, HACS) and leads to substantial nutritional improvements. HMT remains the most dependable single-step physical modification across diverse substrates when the primary goal is glycemic-response attenuation via the conversion of RDS to SDS rather than maximizing absolute RS	[35,58,71,73]
Autoclaving	70–90% moisture; 120–150 °C, 1.5–3.0 atm; 30–120 min/cycle; 1–5 heating–cooling cycles + retrogradation	Median ΔRS +76.8% (range −88.2% to +28,500% *, *n* = 95); median RDS −8.5% (*n* = 26), SDS −23.9% (*n* = 26); strongest RS enhancer overall	High-moisture gelatinization followed by cooling/retrogradation drives extensive amylose leaching and re-association into stable double helices. Repeated cycles amplify this effect in amylose-containing starches, though excess heat or cycling can instead disrupt crystallites already formed.	For waxy starches, hydrothermal treatments generally result in limited RS enrichment, with RS content decreasing systematically as the number of autoclaving–cooling cycles increases. In contrast, increasing cycle number significantly enhances RS formation in common/moderate-amylose starches, while high-amylose starches can achieve substantial RS gains after a single cycle.	[27,32,53,79]

* Percentage changes are calculated relative to each study’s native-starch control and should not be interpreted as direct rankings of process efficiency, due to differences in RS assays, drying procedures, moisture basis, and starch sources across studies (see Section 3.2).

While this review offers a wide overview of studies, one cannot avoid identifying some central internal and external limitations that challenge this and future comparisons between studies. First, the current review mined several articles through the website https://automeris.io/wpd/ (accessed during November 2025) to extract RDS, SDS, and RS values from graphs, as the values were not provided in table form and had to be extracted directly from the figures. That computational tool enabled us to address a larger cohort of studies; however, its accuracy depends both on the computational tool as well as the operator, thus limiting the power of the analysis.

On top of this, two external thematic limitations were identified: one is the methods to obtain starch fractions, and another is the analytical means to determine starch fractions, namely, protocols to determine RS. Most studies mainly involved drying and pulverizing samples and starch fractions that can impact the determination of balances between RDS, SDS, and RS. Notably, samples that are oven-dried often contain more RS than those that are freeze-dried (lyophilized). This effect is attributed to the high temperatures used during oven drying (e.g., ~50 °C compared to freeze-dry), which enable sufficient molecular mobility for retrogradation to continue during the drying stage. In contrast, freeze-drying effectively freezes the starch structure in place, restricting chain movement and limiting further interactions and crystallization [30,93].

Beyond the effect itself, differences in drying procedures can also make it difficult to compare different studies. Oven drying is typically performed at 40–60 °C for 12–24 h until constant weight, conditions that may allow starch chains to remain mobile and continue to retrograde during drying. In contrast, freeze-drying typically involves pre-freezing at −20 to −80 °C (impacting the speed of freezing and lowering chain mobility) followed by vacuum sublimation for 24–72 h. As these methods are not fully reported in all the literature, there is a need to establish a standardized drying protocol or at least unify reporting standards.

Secondly, overviewing the body of evidence echoed a clear gap in methods used to determine RS content and overall starch digestibility, as also identified by others [24]. These studies reveal variations in processing temperatures, durations and specific enzymes used (and their concentrations) in experiments attempting to mimic digestion. This lack of analytical harmonization may dramatically compromise the comparability of findings and generate inconsistencies and confusion. In fact, Polesi et al. [94] provides a direct comparison of how these methods drastically affect the calculated RS content of the same autoclaved chickpea starch samples. Aparicio-Saguilán et al. also noted that RS values determined using Novelose 330 were lower when measured using the TDF method than those obtained using the Goni method [83]. Thus, there is a clear need for methodological harmonization to help converge and disentangle scientific efforts.

## 4. Conclusions

This review offered a targeted overview on the effects of key starch physical modification techniques—ANN, HMT, and autoclaving—on starch digestibility metrics, specifically, digestible RDS and SDS fractions as well as RS. Further, the work demonstrated the use of 21st century tools for an exploratory evaluation of a number of studies through the combined use of human-driven evidence search with an AI-assisted identification of recurring research dimensions and qualitative knowledge extraction. This review highlighted the power of AI to offer a fast and broad overview of open-access literature which was complementary and coincided with a concomitant human-driven search. Collectively, this gathered and significant body of literature supports the notion that modulation of starch digestibility is governed by a delicate balance between structural starch disruption and molecular reassembly of amylose and amylopectin, strongly influenced by intrinsic factors such as botanical origin and amylose content, and extrinsic processing parameters including temperature, moisture, duration, cycle number, and retrogradation conditions. Crucially, the use of AI as a tool in the hands of unsavvy food professionals seeking to underpin qualitative patterns and catalyze conceptual sorting highlighted its strong advantages alongside limitations that warrant a need for careful and responsible use.

From an applied processing perspective, raw material selection should guide protocol choice because starch composition strongly influences treatment outcomes. For high-amylose starches (e.g., Hylon V, Hylon VII, and HACS-55/70), even a single autoclaving heating–cooling cycle already yields marked RS gains owing to their inherent tendency to form stable double-helical amylose structures [29,31,53]. Additional cycles or temperatures above approximately 120–125 °C may provide diminishing or even negative returns because excessive heat can disrupt newly formed crystallites [53]. Therefore, milder HMT conditions are often more resource-efficient for these starches [35,73]. For common starches, using multiple autoclaving–cooling cycles (typically 1–5) followed by retrogradation provides a reliable method for substantial RS enrichment, with RS levels generally increasing as the number of cycles increases [32,33,54]. However, longer autoclaving does not necessarily produce additional RS. Likewise, the optimal retrogradation duration depends on temperature, meaning that longer processing or storage does not inherently increase RS formation [27]. In HMT, higher moisture levels generally favor RS formation although the effects on SDS and RDS are not consistent across studies. During Annealing (ANN), longer treatment times generally promote RS formation. In fact, processing at a temperature approximately 10 °C below the onset temperature of gelatinization (To) has been reported to increase RS content [41], while higher moisture levels may further enhance RS formation [59]. In contrast, all processing methods have relatively limited impact on the RS content of waxy (low/near-zero amylose) starches that contain little or no amylose. Moreover, in such starches increasing processing intensity (i.e., cycles and/or duration) or moisture level may compromise RS levels owing to the intrinsic limitations of amylopectin to reorganize into RS double helices during retrogradation [32,66]. Thus, such starches may benefit more greatly from pairing physical modification with a subsequent chemical or enzymatic treatment.

Overall, autoclaving appears to have the greatest potential for increasing RS when processing conditions are well adapted to the raw material. HMT and ANN can also be useful alternatives for altering the RDS:SDS balance. However, as summarized in Table 4, the magnitude and direction of the effects can vary considerably depending on the amylose content of the native starch. Therefore, it cannot be assumed that the effects observed for one starch type are universally transferable to other raw materials. Importantly, this review highlights that inter-study comparability remains a challenge due to inconsistent analytical methodologies and heterogeneous reporting standards. This limitation is particularly evident in RS research, where variation in analytical protocols and the lack of methodological consensus across quantification approaches compromise the comparability and physiological interpretation of results. Accordingly, the need to develop harmonized methodologies and standardized reporting protocols for RS quantification emerges as a priority area for future research. This need is heightened when seeking to adopt AI-assisted tools to accelerate literature identification and mapping of trends; however, it is important to note that the use of AI tools should be made carefully to circumvent AI limitations to critically evaluate experimental nuances, address methodological variability, and deduce mechanistic insights. Moving forward, supervised and more sophisticated AI-based analyses could help shed new light on the old paradigm of starch modification and transition from empirical “cook and look” schemes toward rational, design-oriented starch engineering. Such 21st century progress is essential to meet aspirations for clean-label processing strategies, enhancing RS intake, and responsible evidence-based innovations.

## Data Availability

The data presented in this study are available on request from the corresponding author due to the size, diversity and complexity of the data.

## Supplementary Materials

The following supporting information can be downloaded at: https://www.mdpi.com/article/10.3390/foods15173137/s1, Table S1: Annealing process parameters, Table S2: HMT process parameters, Table S3: Autoclaving process parameters.

## Abbreviations

The following abbreviations are used in this manuscript:

AI	Artificial intelligence
ANN	Annealing
HMT	Heat–moisture treatment
RDS	Rapidly digestible starch
SDS	Slowly digestible starch
RS	Resistant starch
RS3	Resistant starch type III
USD	United States Dollar
ISI	Institute for Scientific Information
LLMs	Large language models
DOIs	Digital Object Identifiers
AOAC	Association of Official Analytical Chemists
HACS	High-amylose corn starch
Tg	Glass transition temperature
To	Onset temperature of gelatinization
YM	Sweet potato starch Yulmi
YHM	Sweet potato starch Yeonwhangmi
SSPS	Sweet potato starch from Samyang Genex
CSPS	Commercial sweet potato starch
TBS	Tartary buckwheat starch
SS	Sorghum starch
N/A	Not applicable/not available
PF	Tsai-ou lotus rhizome starch
WS	Shih-lian lotus rhizome starch
RMS	Regular maize starch
G50	Gelose 50 high-amylose maize starch
MPG87	Wrinkled pea starch
CDC 4140-4	Wrinkled pea starch
NS	Normal corn starch
HS	Hylon VII
KDML105	Non-waxy rice
RD6	Waxy rice
SR	Rice starch
NCS	Normal amylose starch
HACS-55	High-amylose corn starch, 55% amylose
HACS-70	High-amylose corn starch, 70% amylose
HAM	High-amylose maize
TDF	Total dietary fiber
