# Supplementary material for: Physical Modification of Starch Digestibility: A Comprehensive Review and AI-Assisted Qualitative Knowledge Extraction
Source: Foods. 2026 Sep 3;15(17):3137. doi: 10.3390/foods15173137 (PMC13565489; doi:10.3390/foods15173137)
Supplement: Supplementary file 1 [file foods-15-03137-s001.zip › Supplement 1_tracked revised .pdf]

Table S1: Annealing process parameters.

Starch	Native Amylose content	Pre-treatment	Annealing methodology			Cycles	Retrogradation conditions	RS content evaluation	Num. of replic.	Drying method
			Moist.	Temp.	Duration					
Black 8025 bean starch	25.13ef	N/A	66.7%	50 °C	24 h	1	N/A	In vitro starch digestibility of native and modified bean starches was analyzed using the method described by Englyst et al. (1992).	NA	Dried at 30 °C for 48 h
Pinto Durango bean starches (Simsek et al. 2012)	25.21de									
Castanopsis sclerophylla starch (Shi et al. 2021)	31.11	Equilibrated at constant temperature for 24 h	66.7%	50 °C	24 h	1	N/A	The digestibility of native and annealed starch samples was determined following the procedure described by Englyst and Ye (1992).	3	Air-dried at 40 °C for 24 h
				50 °C	48 h	1			3	
				First step 50 °C second step 55 °C	24 h for each step	2			3	
Sweet potato starch Yulmi	27.30	Stored at room temperature for 24 h for equilibration	90%	55 °C	72 h	1	N/A	Starch digestibility was determined following the method of Brumovsky and Thompson with slight modifications (2001).	3	Air-dried at room temperature
Sweet potato starch Yeonwhangmi	26.60					1			3	
Sweet potato starch from Samyang Genex	26.50					1			3	
Commercial sweet potato starch (Song et al. 2014)	26.60					1			3	

Table S1: Annealing process parameters.

Starch	Native Amylose content	Pre-treatment	Annealing methodology			Cycles	Retrogradation conditions	RS content evaluation	Num. of replic.	Drying method
			Moist.	Temp.	Duration					
Tartary buckwheat starch (TBS)	29.1±0.21b	Equilibration at 4°C overnight	80%	50 °C	24 h	1	N/A	The in vitro starch digestibility was assessed using the classical method reported by Goni, et al. (1996)	3	Air-dried at 40 °C for 12 h.
Sorghum starch (SS) (Liu et al. 2016)	28.7±0.14b					1			3	
PF—Tsai-ou lotus rhizome starch	18.38±0.03ab	Equilibrate overnight at 4°C	80%	50 °C	24 h	1	N/A	RS content of native and modified starch samples was measured using the Resistant Starch Assay Kit protocol (Megazyme International Ireland Ltd., Wicklow, Ireland).	3	Hot air-dried at 40 °C until the moisture content was less than 10% (d.b.).
WS—Shih-lian lotus rhizome starch (Yeh et al. 2021)	16.43 ±0.45a					1			3	
Pea starch	38.2 ± 0.2c	N/A	70%	50 °C	24 h	1	N/A	Enzyme hydrolysis of native and modified pulse starches was determined following the method described by Englyst et al. (1992) with modifications.	2	Air-dried at 40 °C.
Lentil starch	38.9 ± 0.3b					1			2	
Navy bean starch (Chung et al. 2010)	40.8 ± 0.4a					1			2	
Corn starch	24.6 ± 0.3	N/A	70%	52.3 °C	24 h	1	N/A	In vitro starch digestibility was	2	

Table S1: Annealing process parameters.

Starch	Native Amylose content	Pre-treatment	Annealing methodology: Moist.	Annealing methodology: Temp.	Annealing methodology: Duration	Cycles	Retrogradation conditions	RS content evaluation	Num. of replic.	Drying method
				57.3 °C		1		determined following the method described by Englyst et al. (1992) with modifications.	2	
Pea starch	35.4 ± 0.3			44.1 °C		1			2	
				49.1 °C		1			2	Air-dried at room temperature
Lentil starch (Chung et al. 2009)	36.9 ± 0.2			48.1 °C		1			2	
				53.1 °C		1			2	
Grass pea starch Derek	39.0 ± 0.9a	N/A	75%	52.8 °C	24 h	1	N/A	In vitro starch digestibility was determined following the method described by Englyst et al. (1992) with modifications of Chung et al. (2009).	3	Air-dried at room temperature
Grass pea starch Krab (Piecyk et al. 2018)	38.0 ± 0.7a			51.7 °C		1			3	
Highland barley starch (Gu et al. 2025)	23.47±1.52d	N/A	60%	50 °C	12 h	1	N/A	The determination of in vitro digestibility was carried out according to the method by Wang et al. (2018) with some minor adjustments.	3	Dried overnight at 40 ◦C in an air oven
					24 h	1			3	
Yam Dioscorea alata starch	26.58 ± 0.17e	N/A	~67%	50 °C	24 h	1	N/A	The starch digestibility was determined by	3	Cooled to ambient
			80%			1			3	

Table S1: Annealing process parameters.

Starch	Native Amylose content	Pre-treatment	Annealing methodology			Cycles	Retrogradation conditions	RS content evaluation	Num. of replic.	Drying method
			Moist.	Temp.	Duration					
Yam Dioscorea esculenta starch (Bora et al. 2023)	21.34± 0.56h		~67%<br>80%			1<br>1		following the method of Huang et al. (2016) with slight modifications.	3<br>3	temperature and dried at 40 °C for 24 h
Rice starch<br>Maize starch<br>Potato starch<br>(Rosa-Mill´an 2017)	N/A	Equilibrated at 25°C for 4h.	90%	55 °C	12 h	1<br>1<br>1	N/A	The in vitro starch digestibility was determined according to the Englyst et al. (1992) protocol with slight modifications.	3<br>3<br>3	Vacuum-dried at 40 °C for 24 h
Canna starch (Lan et al. 2016)	30.88	N/A	90%	60 °C	1 h<br>3 h<br>5 h<br>7 h	1<br>1<br>1<br>1	N/A	According to Muir method (1995)	N/A	Aliquots of suspensions were taken at different time intervals, no need to dry
Waxy rice starch (Zeng et al. 2015)	0	N/A	80%	50 °C	24 h	1	N/A	Starch nutrition fractions were analyzed according to Englyst et al. (1992) with minor modifications.	2	Air dried at 40°C overnight
Kithul (Caryota urens) starch (Sudheesh et al.2020)	38.55	N/A	75%	55 °C	24 h	1	N/A	The digestibility and RDS, SDS and RS were determined according to procedures of Englyst et al. (1992).	3	N/A

Table S1: Annealing process parameters.

Starch	Native Amylose content	Pre-treatment	Annealing methodology			Cycles	Retrogradation conditions	RS content evaluation	Num. of replic.	Drying method
			Moist.	Temp.	Duration					
Common buckwheat starch (Liu et al. 2015)	28.1 ± 0.23c	Equilibrate overnight at 4 ◦C	80%	50 °C	24 h	1	N/A	The RS content was calculated by a procedure reported by Go˜ni et al. (1996) with modification.	3	Air-dried at 40 °C for 12h
Kithul palm starch (Sudheesh et al.2019)	38.55±0.05d	N/A	80%	50 °C	24 h	1	N/A	The in vitro starch digestibility was determined by using the method proposed by Englyst et al. (1992)	3	Freeze-dried
Water caltrop starch (Liu et al. 2021)	23.29±0.90ab	N/A	70%	50 °C	24 h	1	N/A	In vitro digestibility was determined using the Megazyme resistant starch kit (K-RSTAR) following Englyst et al. (1992) and AOAC 2002.02/AACC 32-40.01, with minor modifications**.**	3	Dried at 40 °C until the moisture content was less than 10% (d.b.)
Chinese yam (Dioscorea opposita Thunb.) starch (Yu et al. 2021)	N/A	N/A	70%	50 °C	24 h	1	N/A	The starch digestibility was determined by the partially modified method of Englyst et al. (1992)	3	Dried at 40 °C.
Native quinoa starch (Liu et al. 2026)	25.40±0.35d	Equilibrate overnight at 4 °C	80%	50 °C	24 h	1	N/A	In vitro digestibility was determined as described by Liu et. al. (2016).	3	Air-dried at 40 °C

Table S1: Annealing process parameters.

Starch	Native Amylose content	Pre-treatment	Annealing methodology			Cycles	Retrogradation conditions	RS content evaluation	Num. of replic.	Drying method
			Moist.	Temp.	Duration					
Corn starch (Liang et al. 2020)	N/A	N/A	43%	50 °C	12 h	1	N/A	Englyst method (1999) with the use of GOPOD assay kit, Megazyme International Ltd. Co.	3	Dried at 50 °C overnight
Bitter vetch (vicia ervilia) starch (Tarahi et al. 2025)	32.87±1.22	Equilibration at 4 °C for 24 h	70%	50 °C	24 h	1	N/A	The in vitro starch digestibility of starch samples was determined following a procedure described by Englyst et al. (1992) with some modifications.	3	Air-dried at 38±2 °C for 24 h.
White sorghum starch (Giuberti et al. 2019)	N/A	N/A	80%	50 °C	24 h	1	N/A	A commercial RS assay kit (K-RSTAR 02/17; Megazyme) was used to quantify RS	3	Oven-dried at 40 °C for 24 h to reach 9–10% moisture
