# Supplementary material for: Physical Modification of Starch Digestibility: A Comprehensive Review and AI-Assisted Qualitative Knowledge Extraction
Source: Foods. 2026 Sep 3;15(17):3137. doi: 10.3390/foods15173137 (PMC13565489; doi:10.3390/foods15173137)
Supplement: Supplementary file 1 [file foods-15-03137-s001.zip › Supplement 2_tracked revised .pdf]

Table S2: HMT process parameters.

Starch	Native Amylose content	Pre-treatment	HMT methodology Moist.	Temp.	Duration	Cycles	Retrogradation conditions	RS content evaluation	Num. of replic.	Drying method
Rice starch (Wang et al. 2018)	13.50	Equilibrated at 4 °C for 24 h.	10%<br>20%<br>30%	110 °C	4 h	1	N/A	In vitro starch digestibility was analyzed with a modified Englyst procedure (1992).	3<br>3<br>3	Dried at 40 °C until sample moisture reached ~11%.
Red Adzuki bean starch (Wang et al. 2017)	N/A	N/A	10%<br>15%<br>20%<br>25%<br>30%	110 °C	4 h	1	N/A	Starch digestibility was determined using a modified Englyst et al. method (1992).	N/A<br>N/A<br>N/A<br>N/A<br>N/A	Dried overnight at 40 °C
Rice starch<br>Maize starch<br>Potato starch (Rosa-Mill´an 2017)	N/A	Equilibrated at 4 °C for 4 h.	30%	120 °C	12 h	1	N/A	In vitro starch digestibility was assessed following the Englyst et al. (1992) protocol with slight modifications.	3<br>3<br>3	Vacuum-dried at 40 °C for 24 h
Sweet potato starch (Sun et al. 2023)	27.73±0.85	Equilibrated at 4 °C for 24 h.	25%<br>25%<br>25%	100 °C<br>110 °C<br>120 °C	4 h	1	N/A	In vitro digestibility of starch samples was determined by	3<br>3<br>3	Dried in an oven at 45 °C for 24 h

Table S2: HMT process parameters.

Starch	Native Amylose content	Pre-treatment	HMT methodology			Cycles	Retrogradation conditions	RS content evaluation	Num. of replic.	Drying method
			Moist.	Temp.	Duration					
			25%	130 °C				the Englyst method (1992).	3	
Jackfruit seed starch (Vu et al. 2024)	N/A	Equilibrated at 10 °C for 15 h.	20%	110 °C	16 h	1	N/A	RS, RDS and SDS contents were determined using a Megazyme RS Assay Kit (AOAC Method 2002.02).	3	Dried at 50 °C for 4 h
			25%	110 °C					3	
			30%	110 °C					3	
Regular maize starch (RMS)	19.10	Equilibrated at 4 °C for 24 h	20%	120 °C	2 h	1	N/A	The in vitro digestibility was determined using a modified Englyst et al. method (1992).	3	Dried at 40 °C for 12 h
			25%						3	
			30%						3	
Gelose 50 high-amylose maize starch (G50) (Wang et al. 2016)	51.60		20%						3	
			25%						3	
			30%						3	
Wrinkled pea starch (MPG87)	74.20	Equilibrated at room temperature overnight	35%	110 °C	6 h	1	N/A	In vitro digestibility starch samples were determined using the Englyst et al. method (1992) with minor changes.	3	Drying at 40 °C overnight.
				130 °C					3	
Wrinkled pea starch (CDC 4140-4)	76.50			110 °C					3	
				130 °C					3	
Round pea starch (Terese)	35.90			110 °C					3	
				130 °C					3	
Round pea starch (CDC Bronco)	34.80			110 °C					3	
				130 °C					3	

Table S2: HMT process parameters.

Starch	Native Amylose content	Pre-treatment	HMT methodology			Cycles	Retrogradation conditions	RS content evaluation	Num. of replic.	Drying method
			Moist.	Temp.	Duration					
(Cheng et al. 2024)										
Water caltrop starch (Liu et al. 2021)	23.29±0.90ab	N/A	20%	105 °C	16 h	1	N/A	Megazyme K-RSTAR kit, based on Englyst (1992) and AOAC 2002.02/AACC 32-40.01 methods with modifications.	3	N/A
			25%			1			3	
			30%			1			3	
PF - Tsai-ou lotus rhizome starch	18.38±0.03ab	Equilibrate overnight at 4 °C	20%	105 °C	16 h	1	N/A	RS content was determined using the analysis procedure provided by the Megazyme Resistant Starch Assay Kit (Wicklow, Ireland).	3	Dried at 40 °C until moisture was less than 10% (d.b.).
			25%			1			3	
			30%			1			3	
WS - Shih-lian lotus rhizome starch (Yeh et al. 2021)	16.43 ±0.45a		20%			1			3	
			25%			1			3	
			30%			1			3	
Common buckwheat starch (Liu et al. 2015)	28.1 ± 0.23c	Equilibrate overnight at 4 °C	20%	110 °C	16 h	1	N/A	The RS content was calculated by a procedure reported by Go˜ni et al. (1996) with modifications.	3	Air-dried at 40 °C for 12 h
			25%			1			3	
			30%			1			3	
			35%			1			3	
Pea	38.2 ± 0.2c	Kept for 24 h at ambient temperature	30%	120 °C	24 h	1	N/A	In vitro starch digestibility was determined using a	2	Air-dried to uniform moisture
Lentil	38.9 ± 0.3b					1			2	

Table S2: HMT process parameters.

Starch	Native Amylose content	Pre-treatment	HMT methodology Moist.	Temp.	Duration	Cycles	Retrogradation conditions	RS content evaluation	Num. of replic.	Drying method
Navy bean (Chung et al. 2010)	40.8 ± 0.4a					1		modified Englyst et al. (1992) method.	2	content (10%).
Grass pea starch Derek	39.0 ± 0.9a	Kept for 24 h at ambient temperature	20%	120 °C	2 h	1	N/A	In vitro starch digestibility was determined following the method described by Englyst et al. (1992) with modifications of Chung et al. (2009).	3	Starch samples were air dried.
Grass pea starch Krab (Piecyk et al. 2018)	38.0 ± 0.7a					1			3	
Corn	24.6 ± 0.3	N/A	30%	100 °C	2 h	1	N/A	In vitro starch digestibility was determined following the method described by Englyst et al. (1992) with modifications.	2	Air-dried at room temperature
				120 °C		1			2	
Pea	35.4 ± 0.3			100 °C		1			2	
				120 °C		1			2	
Lentil (Chung et al. 2009)	36.9 ± 0.2			100 °C		1			2	
				120 °C		1			2	
Highland barley starch (Gu et al. 2025)	23.47 ± 1.52d	Equilibration at 4 °C for 7 d	20%	110 °C	4 h	1	N/A	In vitro digestibility was determined following the method of Wang et al. (2018) with some minor adjustments.	3	Dried overnight at 40 °C in an air oven
			25%			1			3	
			30%			1			3	
Yam Dioscorea alata starch	26.58 ± 0.17e	Equilibration at 4 °C for 24 h	20%	110 °C	3 h	1	N/A	Starch digestibility was determined using a slightly	3	Dried at 40 °C overnight
			30%	110 °C		1			3	
	21.34 ± 0.56h		20%	110 °C		1			3	

Table S2: HMT process parameters.

Starch	Native Amylose content	Pre-treatment	HMT methodology			Cycles	Retrogradation conditions	RS content evaluation	Num. of replic.	Drying method
			Moist.	Temp.	Duration					
Yam Dioscorea esculenta starch (Bora et al. 2023)			30%	110 °C		1		modified Huang et al. (2016) method.	3	
Corn starch (Liang et al. 2020)	N/A	Equilibrate for 24 h at 25 °C	30%	100 °C	12 h	1	N/A	The in vitro starch digestion rate was evaluated using a modified Englyst (1999) method with the GOPOD assay kit (Megazyme International Ltd.).	3	Dried at 50 °C overnight
Banana starch (Sun et al. 2025)		Equilibrate at 25 °C for 24 h	30%	120 °C	1 h	1	N/A	The simulated digestion of starch was determined according to the method described by Ye et al. (2019).	3	N/A
Rice starch (Xie et al. 2020)	N/A	Equilibrated at 4 °C for 24 h	30%	110 °C	4 h	1	N/A	Starch digestibility was measured based on Englyst et al.'s method (1992) with some modifications.	3	Air-dried at 40 °C for 12h
Talipot starch (Aaliya et al. 2021)	28.81 ± 0.01f	Equilibrated for 24 h at 4±2 °C	30%	110 °C	12 h	1	N/A	RDS, SDS, and RS were determined following the method of Englyst et al. (1992)	3	Overnight in a hot air oven at 40 °C
Proso millet starch (Zheng et al. 2020)	14.7 ± 1.2d	N/A	80%	100 °C	1/2 h	1	N/A	A K-RSTAR kit (Megazyme) was used to determine	3	Freeze-dried

Table S2: HMT process parameters.

Starch	Native Amylose content	Pre-treatment	HMT methodology			Cycles	Retrogradation conditions	RS content evaluation	Num. of replic.	Drying method
			Moist.	Temp.	Duration					
								the resistant starch content.		
Kithul (Caryota urens) starch (Sudheesh et al. 2020)	38.55	N/A	N/A	100 °C	16 h	1	N/A	The digestibility of kithul starch samples were found out by the procedures of Englyst et al. (1992).	3	Dried at 45 °C to a moisture level (about 10%).
White sorghum starch (Giuberti et al. 2019)	N/A	Equilibration at 4 °C for 24 h	25%	100 °C	10 h	1	N/A	A commercial RS assay kit (K-RSTAR 02/17; Megazyme) was used to quantify RS.	3	Oven-dried at 40 °C for 24 h to 9–10% moisture.
Waxy rice starch (Zeng et al. 2015)	0	Equilibration at 4 °C for 12 h	25%	110 °C	8 h	1	N/A	Starch nutrition fractions were analyzed according to the method of (Englyst et al., 1992) with minor modifications.	2	Air-dried to uniform moisture content (about 12%)
Yam starch (Yu et al. 2021)	N/A	Equilibration at 4 °C for 24 h	20%	100 °C	10 h	1	N/A	The starch digestibility was determined by the partially modified method of Englyst et al. (1992).	3	Dried at 40 °C.
Bitter vetch (vicia ervilia) starch (Tarahi et al. 2025)	32.87±1.22	Equilibration at 4 °C for 24 h	30%	120 °C	24 h	1	N/A	The in vitro starch digestibility was determined following the modified method of Englyst et al. (1992).	3	Air-dried at 38± 2 °C for 24 h.
