# Supplementary material for: Physical Modification of Starch Digestibility: A Comprehensive Review and AI-Assisted Qualitative Knowledge Extraction
Source: Foods. 2026 Sep 3;15(17):3137. doi: 10.3390/foods15173137 (PMC13565489; doi:10.3390/foods15173137)
Supplement: Supplementary file 1 [file foods-15-03137-s001.zip › Supplement 3_tracked revised .pdf]

Table S3: Autoclaving process parameters.

Starch	Native Amylose content	Pre-treatment	Autoclave methodology			Cycles	Retrogradation conditions	RS content evaluation	Num. of replic.	Drying method
			Moist.	Temp.	Duration					
Normal corn starch (NS)	27	Heated at 60°C for 15min under continuous stirring	95%	105 °C	1/2 h	1	N/A	Following the AACC method 32-40 using the kit from Megazyme (K-RSTAR 08/18).	2	Dried in an oven at 40 °C for 24 h
				120 °C		1				
				135 °C		1				
Hylon VII (HS) (Soler et al. 2020)	70			105 °C		1				
				120 °C		1				
				135 °C		1				
Cilacap breadfruit starch (Rahayu et al. 2023)	31.01±0.24d	N/A	80%	100 °C	1/2 h	3	Cooled at RT for 1 h and then stored at 4 ºC for 24 h	The RS content was analyzed by the Goñi et al. (1996) method with modification	3	Dried in a cabinet dryer at 45 °C for 24 h
				121 °C		3				
				140 °C		3				
				100 °C		3	Cooled at RT for 1 h and then stored at 4 ºC for 48 h			
				121 °C		3				
				140 °C		3				
Waxy corn starch (Sung et al. 2020)	N/A	N/A	80%	121 °C	1/2 h	1	Cooled down to 30 °C and stored at 4 °C for 5 days	RDS, SDS, and RS were measured using the authors' method, defined as glucose released at 10 min, between 10 and 240 min, and remaining after 240 min, respectively.	N/A	Freeze-dried
						2				
						3				

Table S3: Autoclaving process parameters.

Starch	Native Amylose content	Pre-treatment	Autoclave methodology			Cycles	Retrogradation conditions	RS content evaluation	Num. of replic.	Drying method
			Moist.	Temp.	Duration					
Cowpea starch (Ratnaningsih et al. 2020)	37.47±0.80c	Pre-heated for 15 min	75%	121 °C	1/3 h	1	4°C for 24h	Resistant starch content was analyzed by method of Goni, et al. 1996.	3	Dried at 50 °C for 24 h
						3			3	
						5			3	
Non-waxy rice KDML105	9.27± 0.63a	Pre-heated and stirrer at 70 °C for 15 min	75%	121 °C	1/3 h	1	Cooled to 25°C for 20min, and stored at 4°C for 24h	RS content was determined by using the AACC Method 32–40.01, following the recommendations of the resistant starch assay kit (K-RSTAR,Megazyme).	4	Dried at 50 °C for 24 h
						3			4	
						5			4	
Waxy rice RD6 (Sangwongchai et al. 2024)	2.71± 0.36c					1			4	
						3			4	
						5			4	
Rice starch: SR-1	8.56 ± 0.14a	N/A	80%	121 °C	1/2 h	2	4°C for 24h	Resistant starch content was determined using the Megazyme Assay Kit-GOPOD, following the approved AACC method 32-40	3	Oven dried at 45 °C
Rice starch: SR-2	17.85±0.85c					2			3	
Rice starch: Pusa sugandh	13.85±0.28b					2			3	
Rice starch: Jhelum (Ashwar et al. 2016)	24.78±1.21d					2			3	

Table S3: Autoclaving process parameters.

Starch	Native Amylose content	Pre-treatment	Autoclave methodology: Moist.	Temp.	Duration	Cycles	Retrogradation conditions	RS content evaluation	Num. of replic.	Drying method
NCS—Normal amylose starch	20	Preheated in a boiling water bath for 20 min while constantly stirring	80%	125 °C	1/2 h	1	4 °C for 24 h; next, this batch of starch was reheated and refrigerated twice.	RDS, SDS, and RS fractions in cassava starch were determined using the Englyst et al. (1992) method.	3	Dried in an oven at 50 °C
HACS-55 high-amylose corn	55					1			3	
HACS-70 high-amylose corn starch (Li et al. 2025)	70					1			3	
Cassava starch (Onyango et al. 2006)	25.2	N/A	91%	121 °C	1/4 h	1	100 °C for 48 h	Method by Englyst et al. 1992	N/A	N/A
					1/2 h	1				
					3/4 h	1				
					1 h	1				
					1 1/2 h	1				
					1 h	1	-20 °C for 6 h			
						1	-20 °C for 24 h			
						1	-20 °C for 48 h			
						1	4 °C for 6 h			
						1	4 °C for 24 h			
						1	4 °C for 48 h			
						1	30 °C for 6 h			

Table S3: Autoclaving process parameters.

Starch	Native Amylose content	Pre-treatment	Autoclave methodology			Cycles	Retrogradation conditions	RS content evaluation	Num. of replic.	Drying method
			Moist.	Temp.	Duration					
						1	30 °C for 24 h			
						1	30 °C for 48 h			
						1	60 °C for 6 h			
						1	60 °C for 24 h			
						1	60 °C for 48 h			
						1	100 °C for 6 h			
						1	100 °C for 24 h			
						1	100 °C for 48 h			
						1	N/A		3	
						2	N/A		3	
			90%			3	N/A		3	
						3	4 °C for 24 h		3	
Culinary banana starch (Khawas et al. 2017)	34.10±0.02	N/A		120 ^{0}C	1/2 h	3	-20 °C for 24 h	The RS content in the culinary banana starch was determined by an enzymatic method (18th Ed; AOAC International: Washington, DC, 2010.)	3	Freeze-dried
						1	N/A		3	
						2	N/A		3	
			80%			3	N/A		3	
						3	4 °C for 24 h		3	

Table S3: Autoclaving process parameters.

Starch	Native Amylose content	Pre-treatment	Autoclave methodology			Cycles	Retrogradation conditions	RS content evaluation	Num. of replic.	Drying method
			Moist.	Temp.	Duration					
						3	-20°C for 24h		3	
Khai Mod Rin rice starch (Junden et al. 2025)	23.26±0.39b	Gelatinized by heating in a water bath maintained at 85 °C for 30 min	≈91%	135 ^{0}C	1/2 h	1	Without chilling	The in vitro starch digestibility was conducted according to the method described in detail by Goni et al . 1997 and Ma et al. 2024, with modifications	3	Dried in a hot-air oven at 45 °C for 24 h
						1	Chilling performed at 4°C for 12h in each cycle		3	
						2			3	
						3			3	
						4			3	
						5			3	
						1	Chilling performed at 4 °C for 24h in each cycle		3	
						2			3	
						3			3	
Potato starch (Yaskin Harush et al. 2025)	N/A	N/A	90%	121 ^{0}C	1/3 h	1	Cooled to 25°C and stored at 4°C for 24h.	RS content was estimated through the enzymatic digestion of the non-RS fraction,	4	Freeze-dried
							Cooled to 40°C and stored at 40°C for 24h.		4	
Hylon V maize starch	≈55	Cooked by boiling with stirring for 30 min	91%	135 °C	1/2 h	1	N/A	RS contents were measured using a	2	Oven dried at 50 °C,

Table S3: Autoclaving process parameters.

Starch	Native Amylose content	Pre-treatment	Autoclave methodology			Cycles	Retrogradation conditions	RS content evaluation	Num. of replic.	Drying method
			Moist.	Temp.	Duration					
Hylon VII maize starch (Ozturk et al. 2009)	≈70							resistant starch kit (Megazyme) according to Approved Method 32-40	2	Freeze-dried
									2	Oven dried at 50 °C
									2	Freeze-dried
Bean (Phaseolus coccineus) starch-Nata	31.4±0.3b	N/A	95%	121 ⁰C	1/4 h	1	N/A	Following Englyst et al 1992 with modifications of Chung et al. 2009	3	N/A
Bean (Phaseolus coccineus) starch-Karo (Piecyk et al., 2013)	32.6±0.4a								3	
High-amylose corn starch Hylon VII (70%) (Dundar 2013)	70	N/A	80%	140 ⁰C	1/2 h	3	4 °C for 24 h	Resistant starch content was assessed using the Megazyme Resistant Starch Assay Kit, following the approved AACC method 32-40 (AACC, 2009)	3	Oven dried at 45 °C
						3	4 °C for 48 h		3	
						3	4 °C for 72 h		3	
				145 ⁰C		3	4 °C for 24 h		3	
						3	4 °C for 48 h		3	
						3	4 °C for 72 h		3	
Cassava roots starch (Khurshida et al. 2022)	N/A	N/A	80%	120±1 °C	1/2 h	3	4 °C for 24 h	Enzyme resistant starch was evaluated according to Eerlingen method(1993).	N/A	Freeze-dried
			90%							

Table S3: Autoclaving process parameters.

Starch	Native Amylose content	Pre-treatment	Autoclave methodology			Cycles	Retrogradation conditions	RS content evaluation	Num. of replic.	Drying method
			Moist.	Temp.	Duration					
Purple sweet potato starch (Sun et. al 2022)	17.87±0.21c	N/A	65%	120 °C	1/3 h	1	4°C for 24 h	Megazyme Resistant Starch Kit was used to determine the RS.	N/A	Dried at 40 °C for 24 h
Banana starch (Aparicio-Saguilána et al. 2005)	37.0±0.5a	N/A	78%	121 °C	1 h	3	4°C for 24 h	The RS content was measured using the procedure of Goñi et al.	3	Freeze-dried
Talipot starch (Aaliya et al. 2021)	28.81±0.01f	N/A	30%	121 °C	3/4 h	1	N/A	Following the method of Englyst et al. (1992).	3	Overnight in a hot air oven at 40 ^{0}C
High-amylose maize (HAM, Hylon VII) (Li et al. 2018)	N/A	N/A	85%	121 °C	1/2 h	1	Stored for 8 days under temperature cycles of 4°C for 1d and 40°C for 1d	RS content was assessed by using Megazyme RS Assay Kit according to method (AOAC, 2002.02).	3	Convectio n oven kept at 40 ^{0}C for 1 day
Black Tartary buckwheat native starch (Zheng at al. 2023)	12.95 ±0.36	Heated at 95 °C for 15 min	95%	122 °C	1/3 h	1	4°C for 24	RS content was determined according to the procedure of Englyst et al. (1992) with modifications.	N/A	Freeze-drying
Euryale ferox starch (Yang et al. 2021)	20.82±1.08c	Heating at 90 °C in a water bath for 30 min	88%	130 °C	1/2 h	1	Cooled at room temperature for 1h and refrigerated at 4°C for 15h	The authors use their own method.	3	Dried to a constant weight at 50 °C

Table S3: Autoclaving process parameters.

Starch	Native Amylose content	Pre-treatment	Autoclave methodology			Cycles	Retrogradation conditions	RS content evaluation	Num. of replic.	Drying method
			Moist.	Temp.	Duration					
Purple sweet potato starch (Bodjrenou et al. 2024)	21.53±0.37a	Stirring at 80 °C for 15 min, followed by 15 min at 95 °C	70%	121 °C	1/3 h	1	Storing at 4 °C for 24 h	The starch fractions in the samples were determined by the method of Thanh et al. (2017) with some modifications.	3	The resultant was dried at 60 °C for 16h
Proso millet starch (Zheng et al. 2020)	14.7 ± 1.2d	N/A	80%	121 °C	1/2 h	1	N/A	A K-RSTAR kit (Megazyme) was used to determine the RS content.	3	Freeze-dried
Kithul starch (Caryota urens) (Sudheesh et al. 2020)	38.55	Starch was adjusted to 25% and equilibrated at 4 °C for 4 days with intermittent mixing.	25%	110 °C	1 h	1	N/A	The digestibility of kithul starch samples were determined by the procedures of Englyst et al. (1992).	3	Dried at 45 °C for 24 h in a hot air oven
Lotus seed starch (Wang et al. 2024)	N/A	Stirred (450 rpm) at room temperature for 24 h	82%	115 °C	1/6 h	1	N/A	The digestibility of samples was evaluated with reference to the method of Englyst et al.(1992).	3	N/A
Normal rice starch (Li et al. 2020)	28.12±0.04c	The suspension was gelatinized in a boiling water bath for 30 min with constant stirring.	80%	121 °C	1 h	1	4 °C for 24 h	Digestion properties were determined following the method of Li et al. (2019) using the NutraScan GI20.	N/A	Dried at 60 °C for 16 h
Buckwheat starch (Tao et al. 2025)	20.23±0.18d	Pretreated in a water bath at 95 °C for 15 min	80%	121 °C	1/2 h	1	4 °C for 24 h	The digestibility was determined using the methods from Englyst et al. (1992) and Wang et	3	Dried at 60 °C for 12 h

Table S3: Autoclaving process parameters.

Starch	Native Amylose content	Pre-treatment	Autoclave methodology			Cycles	Retrogradation conditions	RS content evaluation	Num. of replic.	Drying method
			Moist.	Temp.	Duration					
								al.(2017), with modifications.		
White sorghum starch (Giuberti et al. 2019)	N/A	N/A	80%	121 °C	1/2 h	2	After the first autoclave cycle, the sample was cooled to room temperature and stored at 4 ⁰C for further 24 h.	RS assay kit (K-RSTAR 02/17; Megazyme) was used for the quantification of RS.	3	Oven-dried at 40 °C for 24 h to 9–10% moisture.
Rice starch (Frasson et al. 2024)	24.55±1.48	Equilibrated at 4 °C for 24 h	≈81%	110 °C	1 h	1	Cool slowly for 24 h.	An in vitro digestion model (Dartois et al. 2010) was used. RDS, SDS, and RS were calculated according to Englyst et al. by using the GOPOD assay.	2	Air circulation oven at 40 °C for 24 h
Banxia starch (Li et al. 2021)	27.86±0.24	N/A	75%	121 °C	1/3 h	1	Cooled to 25 °C and stored at 4 °C for 24 h.	According to the method of Englyst et al.(1992), with modifications using the Glucose Assay Kit.	3	Dried at 60 °C for 24 h
Chinese Yam Starch (Xie et al. 2023)	N/A	Pregelatinized and then cooled to 50 °C	≈95%	105 °C	1/2 h	1	Refrigerated at 4 °C for 24 h	In vitro digestibility was determined following Khurshida et al. (2021), with minor modifications.	3	Freeze-dried

Table S3: Autoclaving process parameters.

Starch	Native Amylose content	Pre-treatment	Autoclave methodology			Cycles	Retrogradation conditions	RS content evaluation	Num. of replic.	Drying method
			Moist.	Temp.	Duration					
Pea starch (Cui et al. 2021)	N/A	Pregelatinized in boiling water for 5 min	83%	121 °C	1/2 h	1	Refrigerated at 4 °C for 24 h	The RS content was quantified using the Megazyme resistant starch assay kit following the AOAC official method 2002.02.	3	Freeze-dried
